# Long non-coding RNA *NEAT1*-modulated abnormal lipolysis via ATGL drives hepatocellular carcinoma proliferation

**DOI:** 10.1186/s12943-018-0838-5

**Published:** 2018-05-15

**Authors:** Xirui Liu, Yingjian Liang, Ruipeng Song, Guangchao Yang, Jihua Han, Yaliang Lan, Shangha Pan, Mingxi Zhu, Yao Liu, Yan Wang, Fanzheng Meng, Yifeng Cui, Jiabei Wang, Bo Zhang, Xuan Song, Zhaoyang Lu, Tongsen Zheng, Lianxin Liu

**Affiliations:** 1 Department of Hepatic Surgery, The First Affiliated Hospital of Harbin Medical University, Key Laboratory of Hepatosplenic Surgery, Ministry of Education, Harbin, Heilongjiang Province China; 2 Key Laboratory of Hepatosplenic Surgery, Ministry of Education, The First Affiliated Hospital of Harbin Medical University, Harbin, Heilongjiang Province China; 3 Department of Gastrointestinal Medical Oncology, The Affiliated Tumour Hospital of Harbin Medical University, Harbin, Heilongjiang China; 4 Department of Pharmacology (the State-Province Key Laboratories of Biomedicine-Pharmaceutics of China, Key Laboratory of Cardiovascular Research, Ministry of Education), Harbin Medical University, Harbin, China

**Keywords:** Hepatocellular carcinoma, *NEAT1*, ATGL/PNPLA2, Lipolysis, LncRNA, Lipid metablosim

## Abstract

**Background:**

Abnormal metabolism, including abnormal lipid metabolism, is a hallmark of cancer cells. Some studies have demonstrated that the lipogenic pathway might promote the development of hepatocellular carcinoma (HCC). However, the role of the lipolytic pathway in HCC has not been elucidated.

**Methods:**

We compared levels of adipose triglyceride lipase (ATGL) in human HCC and healthy liver tissues by real time PCR, western blot and immunohistochemistry. We measured diacylglycerol(DAG) and free fatty acid (FFA) levels in HCC cells driven by the *NEAT1*-ATGL axis and in HCC tissues. We also assessed the effects of ATGL, DAG, FFA, and *NEAT1* on HCC cells proliferation in vitro and in an orthotopic xenograft HCC mouse model. We also performed a luciferase reporter assay to investigate the interaction between *NEAT1*/ATGL and miR-124-3p.

**Results:**

We found that the lipolytic enzyme, ATGL is highly expressed in human HCC tissues and predicts poor prognosis. We also found that high levels of DAG and FFA are present in HCC tissues. Furthermore, the lncRNA-*NEAT1* was found to modulate ATGL expression and disrupt lipolysis in HCC cells via ATGL*.* Notably, ATGL and its products, DAG and FFA, were shown to be responsible for *NEAT1*-mediated HCC cell growth. *NEAT1* regulated ATGL expression by binding miR-124-3p. Additionally, *NEAT1* knockdown attenuated HCC cell growth through miR-124-3p/ATGL/DAG+FFA/PPARα signaling.

**Conclusion:**

Our results reveal that *NEAT1-*modulates abnormal lipolysis via ATGL to drive HCC proliferation.

**Electronic supplementary material:**

The online version of this article (10.1186/s12943-018-0838-5) contains supplementary material, which is available to authorized users.

## Background

Hepatocellular carcinoma (HCC) is the sixth most common cancer worldwide and the second leading cause of cancer death in men [1]. The progression of HCC is characterized by abnormal cell differentiation, fast infiltrating growth, early metastasis, high-grade malignancy, and poor prognosis [2]. Abnormal metabolism, including abnormal lipid metabolism, is a hallmark of cancer cells. Large amounts of FAs are required to accommodate high rates of proliferation in cancer cells [3]. Cancer cells can acquire FAs through lipogenic and lipolytic pathways [4].

Currently, it is believe that adipose triglyceride lipase (ATGL), hormone-sensitive lipase (HSL) and monoacyglycerol lipase (MAGL) are the main enzymes involved in lipolysis [5]. ATGL initiates the process of triglyceride (TAG) metabolism by hydrolyzing TAG into diacylglycerol (DAG) and FFA. The subsequent step requires HSL, which breaks down DAG into monoacylglycerol (MAG). Finally, MAG is further broken down into FFA and glycerol by MAGL. Daniel et al. revealed that the lipolytic enzyme, MAGL promotes migration, survival, and in vivo tumor growth through the MAGL- free fatty acid (FFA) pathway [6]. However, other studies indicated that MAGL acts as a tumor suppressor role [7]. These studies indicate that the role of lipolytic enzymes in tumor progression requires further study. Although, recent studies have revealed that the inhibition of ATGL via RNAi or a small molecule inhibitor attenuated the growth and motility of tumor cells (colorectal cancer cells and non-small cell lung carcinomas cells) [8], However, whether it contributes to tumor growth, or other functions in HCC remains unclear.

Nuclear paraspeckle assembly transcript 1 (*NEAT1*) is a nuclear-enriched lncRNA and is a scaffolding factor that is necessary for the formation of nuclear paraspeckles [9]. *NEAT1* is up-regulated in various types of cancers and has been reported to be associated with unfavorable prognosis in cancer patients [10]. *NEAT1* was demonstrated to function as a competing endogenous RNA (ceRNA) by competitively binding common microRNAs [11, 12]. Although recent studies have demonstrated that *NEAT1* is overexpressed specifically in HCC [13], the mechanism through which *NEAT1* affects tumor progression requires further study.

We hereby report that the lncRNA-*NEAT1* disrupts HCC cell lipolysis through ATGL. Our results explain the high levels of DAG and FFA present in HCC tissues. ATGL and its products, DAG and FFA, are responsible for *NEAT1*-mediated HCC cell growth. Additionally, *NEAT1* mediates HCC cell growth through the miR-124-3p/ATGL/DAG+FFA/PPARα pathway. Thus, we demonstrate that *NEAT1*-mediated abnormal lipolysis promotes HCC cell growth**.**

## Methods

### Patients and tissue samples

From 2008 to 2012, archival HCC tissues were obtained from patients at the First Affiliated Hospital of Harbin Medical University. Informed consent was obtained from each patient prior to biopsy or surgery, and ethical approval for the use of human subjects was obtained from the Research Ethics Committee of the First Affiliated Hospital of Harbin Medical University. Detailed characteristics of patients were summarized in Additional file 1: Table S1 and Table S2.

### Cell lines and culture conditions

The L02 immortalized liver cell line and 293 T was purchased from the Institute of Biochemistry and Cell Biology, Chinese Academy of Science, China. HepG2, Huh7, SK-Hep-1, and HCCLM3 were purchased from the American Type Culture Collection (Manassas, VA, USA). Huh7-luciferase-transfected and HCCLM3-luciferase-transfected cells were purchased from Berthold Technologies. All cell lines were cultured in DMEM supplemented with 10% FBS, 100 units/mL penicillin, and 100 μg/mL streptomycin.

### Lentiviral infection

Human Lenti-sh*NEAT1*-GFP, Lenti-shPNPLA2-GFP, Lenti-PNPLA2-GFP, Lenti-vector-GFP control and Lenti-sh-control-GFP were designed and purchased from GeneChem Technologies (Shanghai, China). Transfection was performed according to standard procedures. The shRNA sequences used in this study are listed as follow:

- NEAT1-shRNA#1:GTGAGAAGTTGCTTAGAAA;
- NEAT1-shRNA#2:TGGTAATGGTGGAGGAAGA;
- ATGL(PNPLA2)-shRNA#1:AAGTTCATTGAGGTATCTA;ATGL(PNPLA2)-shRNA#2:CTTTACTCCTGAGAACTTT.

### Transient transfection

Small interfering RNA (siRNA), si-control, miR-124-3p mimic, miR-124-3p miR-124-3p inhibitor, negative control were purchased from Ribobio (Guangzhou, China). For transfection, miR-124-3p mimic, miR-124-3p inhibitor, negative control, siRNA or si-control in Lipofectamine 2000 (Invitrogen) was transfected into cells according to the manufacturer’s instructions. The siRNA sequences are listed in Additional file 2: Table S3.

### Cell proliferation assay

For the Cell Counting Kit-8 assay (CCK-8), cells were seeded at a density of 2500–4000 cells/well in 96-well plates. In vitro cell proliferation was assessed by Cell Counting Kit-8 (Dojindo) according to the manufacturer’s instructions. For the cell growth assays, HCC cells were seeded at a density of 0.5 × 10^4^ per well. The number of viable cells was determined at different timepoints. For the Colony formation assay, cells were seeded in 6-well plates at a density of 500–800 cells/well and cultured for 14 days. Colonies were stained with 0.5% Crystal Violet for 10 min and counted.

### Measurement of DAG and FFA contents

DAG and FFA contents were determined with a DAG ELISA Kit (BlueGene Biotech, E01D0010) and Free Fatty Acid Quantitation Kit (Sigma, MAK044) respectively, following the manufacturers’ protocols.

### Western blot

Western blot analysis was performed as previously described [14]. In brief, whole cell or tissue extracts were prepared using RIPA buffer. After electrophoresis, proteins were electroeluted at 120 Volts onto a polyvinylidenedifluoride (PVDF) membrane (Invitrogen). Indicated primary antibodies were used. Protein bands were visualized by an enhanced chemiluminescence assay kit (Super Signal Pierce Bio-technology). The following antibodies against ATGL/PNPLA2(Cayman, 10006409), PPARα(Abcam, ab8934), MAGL(Abcam, ab24701), HSL(Abcam,ab45422), p53(Santa Cruz, SC126) and p21(Abcam, ab109520), Bax (Abcam, ab32503), β-Actin (Cell Signaling Technology) were used. Western blot analyses were repeated at least three times.

### Immunohistochemical analysis

Expression of Ki-67 and ATGL was evaluated using an immunohistochemical (IHC) method described previously [14].

### Reagents

1,3-Dilinoleoyl-*rac*-glycerol (Sigma, D9508) was dissolved in fresh dimethyl sulfoxide (DMSO) for stock solution at 50 mM (or 50 mg/ml for the in vivo study). Similarly, oleic acid (Sigma, O1008) was dissolved in fresh DMSO to 50 mM (or 50 mg/ml for the in vivo study). 1,3-Dilinoleoyl-*rac*-glycerol and oleic acid were mixed at a 1:1 ratio to prepare the DAG+FFA mixture. Working solution was added with pre-set DAG and FFA concentrations by mixing common serum-free medium proportionately. Final concentrations were set at 8 μM, 16 μM and 32 μM. Atglistatin (Sigma, SML1075) was dissolved in DMSO for stock solution at 10 mM. Final working concentrations of 40 μM Atglistatin was used in all experiments. Nutlin-3a(Sigma, SML0580) was dissolved in DMSO for stock solution at 5 mg/ml. Final working concentrations of 10 μM Nutlin-3a was used in experiments.

#### Real time PCR analysis

Total RNA was extracted from cultured cells using the RNAeasy Mini kit (Qiagen, Valencia, CA, USA) according to the manufacturer’s instructions. Reverse transcription was performed using the High Capacity Reverse Transcription kit (Applied Biosystems, Foster City, CA, USA) after RNA quantification. Real-time PCR was performed using the Power SYBR Green PCR Master Mix (Life Technologies, Carlsbad, CA, USA) on an ABI Prism 7900HT instrument (Applied Biosystems). Real-time PCR was performed in triplicate. The expression of lncRNA and protein coding genes were normalized to that of β-Actin gene. Specifically, stem-loop reverse transcriptase polymerase chain reaction was used in the analysis of mature miRNA expression. The stem-loop RT primer was CCTGTTGTCTCCAGCCACAAAAGAGCACAATATTTCAGGAGACAACAGGGGCATTC. The reverse transcriptase reactions was performed as described previously [15]. Reverse transcriptase reactions contained 2 μg total RNA, 50 nM stem–loop RT primer, 1× RT buffer (Applied Biosystems, Foster City, CA, USA), 0.25 mM dNTPs, 3.33 U/μl MultiScribe reverse transcriptase (Applied Biosystems, Foster City, CA, USA) and 0.25 U/μl RNase inhibitor (Applied Biosystems, Foster City, CA, USA). The reaction parameters were as follows: 30 min at 16 °C, 30 min at 42 °C, 5 min at 85 °C and then held at 4 °C. Quantitative RT-PCR reaction parameters were as follows: 2 min at 50 °C, 2 min at 95 °C, 40 cycles of denaturation at 95 °C for 30 s, Annealing at 60 °C for 1 min. The expression of miRNA was normalized to that of U6 gene. Expression levels of target genes were determined according to the 2^−ΔΔCt^ method. Primers used are listed in Additional file 2: Table S4.

### Fluorescent In Situ Hybridization (FISH)

Fluorescent In situ hybridization (FISH) was performed with a Fluorescent In Situ Hybridization Kit (RiboBio, Guangzhou, China), following the manufacturers’ protocols. The CY3 labeled miR-124-3p probe were designed and purchased from GenePharma Technologies (Shanghai, China). The sequence was: 5’-GGCAUUCACCGCGUGCCUUA-3′.

### P53 mutational analysis by PCR and direct sequencing

The mutation detection of the p53 gene was carried out by amplification of exons 2–11 from genomic DNA with 7 pairs of primers. Primer sequences are listed in Additional file 2: Table S5. Ex TaqDNA Polymerase used in PCR amplification was purchased from Takara, Japan. Amplification was done following the manufacturers’ protocols. PCR products were sequenced using an ABI3730XL DNA sequencer (Applied Biosystems). Profiles of p53 mutation were showed in Additional file 2: Table S6.

### Animal studies

Male BALB/c nude mice (4–6 weeks old) were obtained from the experimental animal center of the Shanghai Institute for Biological Sciences (SIBS) and housed under standard conditions and care according to the institutional guidelines for animal care. All animal experiments were approved by the Institutional Animal Care and Use Committee of Harbin Medical University. To establish an orthotopic HCC mouse model, 4 × 10^6^ cells in 100 μL of phosphate-buffered saline were subcutaneously injected into the flanks of nude mice. After 1–2 weeks, the subcutaneous tumors were resected and diced into 1 mm^3^ cubes, which were then implanted into the left lobes of the livers of the nude mice. A DAG and FFA mixture was used in vivo experiment. DAG and FFA solution was prepared in 20% DMSO and 15% Tween 80 in 0.9% saline. After 1 week of implantation mice were injected 20 mg/kg DAG+FFA intraperitoneally for 5 weeks (5 days per week). Mice were imaged by the bioluminescence IVIS Imaging System weekly and mice were then sacrificed.

### Luciferase reporter assay

The luciferase reporter assay was performed according to the method described previously [16].

### Statistical analysis

Statistical analysis was performed with the GraphPad Prism software package (v. 4.02; San Diego, CA, USA) or SPSS 16.0 software (Chicago, IL, USA). Student’s t-test or one-way ANOVA was applied to determine the significance between groups. Statistical analyses between different treatments, in different cell cohorts or at different time points were performed using two-way ANOVA with the Bonferroni’s correction. Overall survival (OS) was compared with the Kaplan-Meier method, and the significance was determined by the log-rank test. Correlations were calculated using Spearman rank-order coefficients. Values were expressed as mean ± SD values. Statistically significant was concluded at *P < 0.05, **P < 0.01, ***P < 0.001; NS represents no statistically significant.

## Results

### ATGL is highly expressed in human HCC tissues and predicts a poor prognosis

**Fig. 1:**
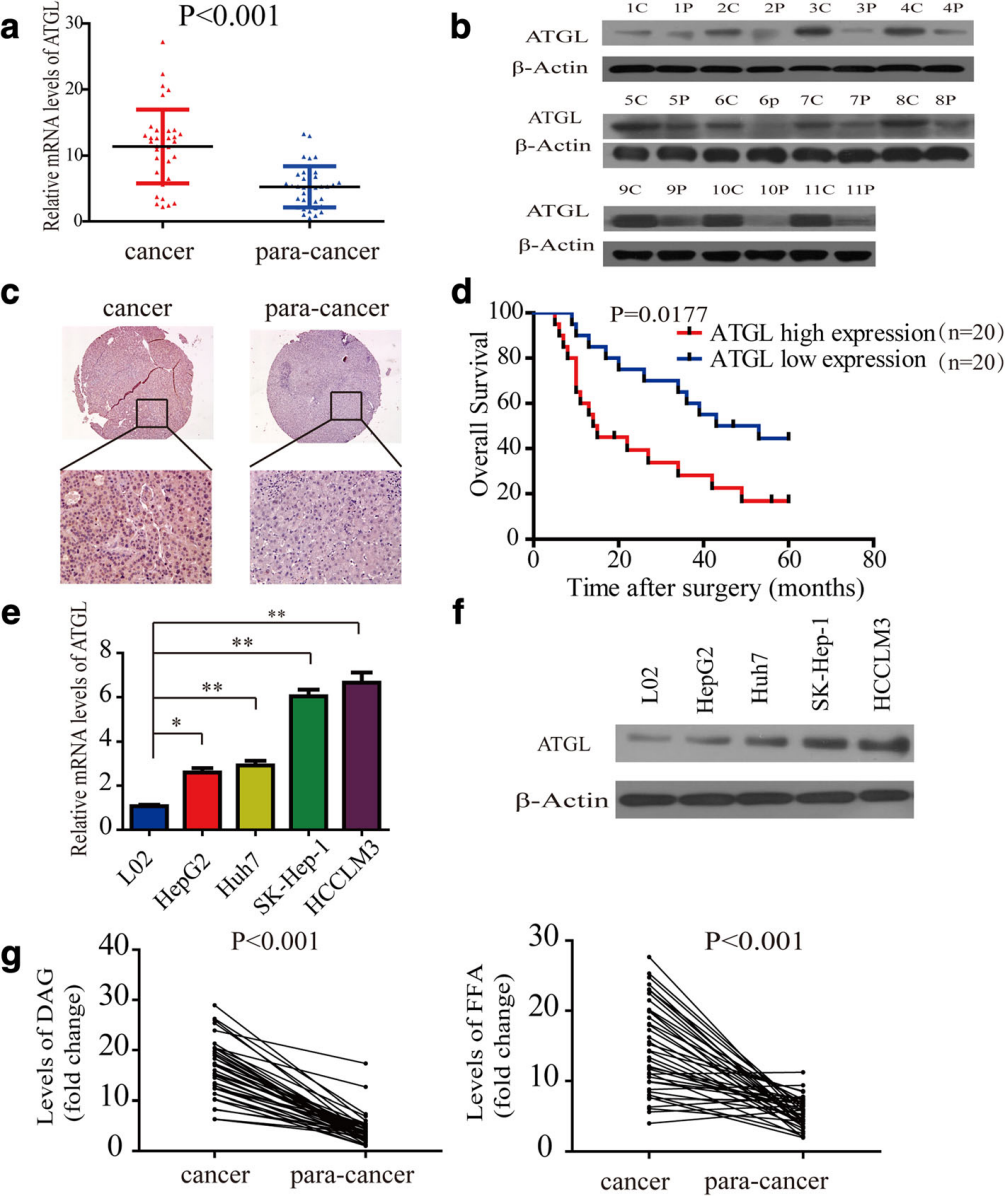
ATGL is highly expressed in human HCC tissues and predicts poor prognosis. **a** Real-time PCR analysis of ATGL expression in 40 pairs of HCC and matched non-tumor tissues. **b** Western blot analysis of ATGL expression in HCC and matched non-tumor tissues C: cancer tissue; P: para-cancer tissue. **c** Representative immunohistochemical detection of ATGL in normal liver tissue and HCC tissues. **d** Kaplan-Meier analysis of overall survival in 40 patients indicated that high expression of ATGL predicts poor prognosis. The cutoff lines to divide into high and low group was median value. **e** Real-time PCR analysis of ATGL expression in HCC cell lines. **f** Western blot analysis of ATGL expression in HCC cell lines. **g** Relative levels of DAG and FFA in 40 pairs of HCC and matched non-tumorous tissues. Data are expressed as mean ± SD of three independent experiments. Statistical significance was concluded at *P < 0.05, **P < 0.01

The intracellular lipolytic pathway is currently known to be mainly regulated by three enzymes: namely, ATGL, HSL and MAGL [5]. Thus, we first elucidated whether these three enzymes were aberrantly expressed in five pairs of HCC tissues and adjacent non-tumorous liver tissues. The results indicated that ATGL mRNA was more aberrantly expressed in HCC tissues than HSL and MAGL mRNA (Additional file 3: Figure S1A). Next, we evaluated ATGL expression in 40 pairs of HCC tissues. Quantitative real-time PCR (qRT-PCR) revealed that mRNA levels of ATGL were higher in HCC tissues than in their adjacent non-tumorous liver tissues (Fig. 1a). Moreover, we found that ATGL expression was positively correlated with tumor size. However, it was not correlated with HBV infection (Additional file 1: Table S1). We also evaluated the expression of ATGL by western blot in clinical HCC tissues. The expression of ATGL was higher in HCC tissues than in their adjacent non-tumorous liver tissues (Fig. 1b). In addition, we evaluated the expression of ATGL by immunohistochemical (IHC) staining in clinical HCC tissues using tissue microarrays and found that 76.11% (86/113) were positive in HCC tissues compared with 12.20% (5/41) in normal liver tissues (Fig. 1c). Moreover, ATGL expression was negatively correlated with patient survival in 40 HCC tissues (Fig. 1d). To elucidate the effect of ATGL on HCC carcinogenesis, we analyzed ATGL protein and mRNA levels in a panel of HCC cell lines. The results indicated that ATGL protein and mRNA levels increased progressively from healthy liver cells to HCC cells with low growth potential and, finally, to HCC cells with high growth potential (Fig. 1e, f).

ATGL has been reported to initiate the process of TAG metabolism by hydrolyzing TAG into DAG and FFA [17]. To confirm this process in HCC cells, we generated lentiviral constructs expressing ATGL-GFP and sh-ATGL-GFP to infect HCC cells. Western blot results confirmed remarkable ATGL overexpression (Additional file 3: Figure S1B). Simultaneously, Compared with the control shRNA, ATGL expression was notably decreased by lenti-sh-ATGL#2 as shown in Additional file 3: Figure S1B. Thus, sh-ATGL-#2 was chosen for further experiments. Our data indicated that overexpression of ATGL increased intracellular FFA and DAG levels in HCC cells (Additional file 3: Figure S1C). Similarly, ATGL knockdown or treatment with Atglistatin (selective inhibitor of ATGL) reduced in intracellular FFA and DAG levels (Additional file 3: Figure S1D). In addition, we found that higher levels of DAG and FFA accumulated in HCC tissues compared with in the corresponding peritumoral tissues (Fig. 1g). These experimental results suggested that ATGL was highly expressed and maintained elevated levels of DAG and FFA in human HCC tissues. This indicated that ATGL and its products, DAG and FFA, may play important roles in HCC development.

### ATGL promotes HCC cell growth by maintaining elevated levels of DAG and FFA in vitro and in vivo

**Fig. 2:**
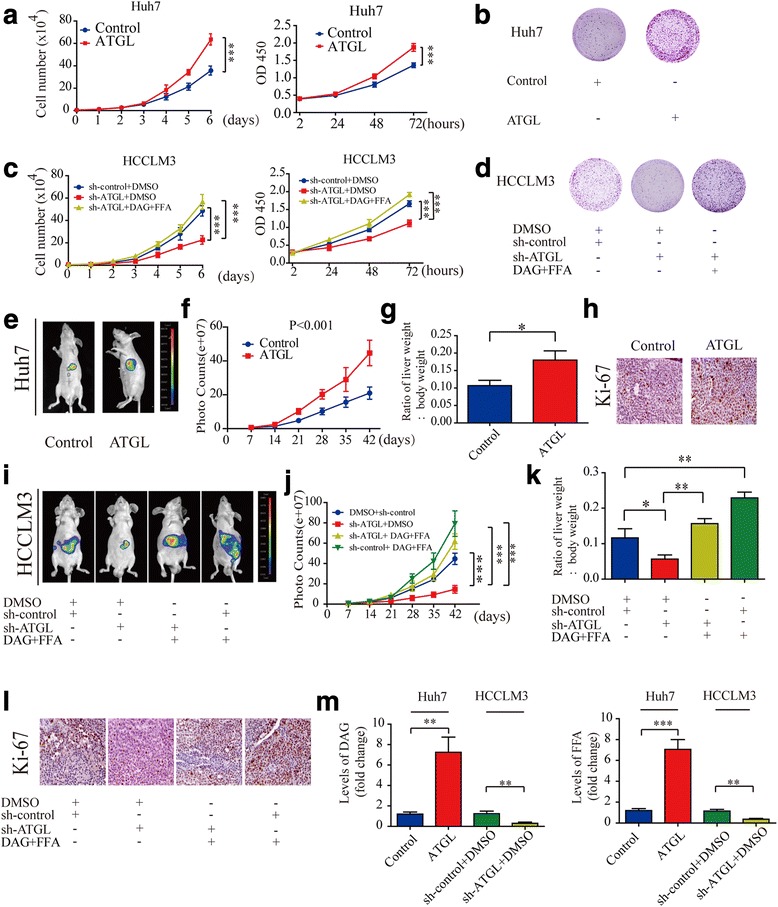
ATGL promotes HCC cell growth by maintaining elevated levels of DAG and FFA in vitro and in vivo*.* **a** Growth curves for the indicated HCC cells were evaluated by the Trypan blue dye exclusion method (left panel). CCK-8 assays showed that overexpression of ATGL promoted the growth of Huh7 cells (right panels). **b** Representative images of the cloning formation assay showed that overexpression of ATGL promoted the growth of Huh7 cells. **c** Growth curves for the indicated HCC cells were evaluated by the Trypan blue dye exclusion method (left panel). CCK-8 assays showed that sh-ATGL significantly inhibited HCC cell growth, however, this effect was completely rescued by treatment with 16 μM DAG+FFA (right panel). **d** Representative images of the cloning formation assay· showed that sh-ATGL significantly inhibited HCC cell growth and this effect was completely rescued by treatment with 16 μM DAG +FFA. **e** Representative bioluminescence imaging of Huh7 orthotopic HCC tumors **f** Volume of Huh-7 orthotopic tumors was determined at different timepoints. **g** The ratio of liver weight/body weight. **h** Representative images of immunohistochemical detection of Ki-67 in orthotopic HCC tissues. **i** Representative bioluminescence imaging of HCCLM3 orthotopic HCC tumors. **j** Volume of HCCLM3 orthotopic tumors was determined at different timepoints. **k** The ratio of liver weight/body weight. **l** Representative images of immunohistochemical detection of Ki-67 in orthotopic HCC tissues. **m** Relative levels of DAG and FFA was determined in 100 mg orthotopic HCC tissues. Two-way ANOVA with the Bonferroni’s correction was used in different timepoints statistical analysis. Data are expressed as mean ± SD of three independent experiments. Statistical significance was concluded at *P < 0.05, **P < 0.01, ***P < 0.001

We next assess the effects of ATGL on the growth of HCC cells. According to growth curves and CCK-8 assay results, the up-regulation of ATGL significantly promoted Huh7 and HepG2 cell growth (FIig.2a and Additional file 4: Figure S2A). Colony formation assay also revealed that up-regulating ATGL expression resulted in more visible colonies compared with control in Huh7 and HepG2 cell lines (Fig. 2b and Additional file 4: Figure S2B and C).

Next we explored whether DAG and FFA facilitates HCC cell growth. CCK-8 assay results showed that treatment with DAG and FFA separately facilitated HCC cell growth in a dose-dependent manner (Additional file 5: Figure S3A and B). Simultaneously, treatment with16μM DAG together with 16 μM FFA (16 μM DAG+ FFA) in HCC cells resulted in a synergistic effect to facilitate HCC cell growth (Additional file 5: Figure S3C).

Considering the important roles of DAG and FFA in the promotion of HCC cell growth, we hypothesized that the pro-tumorigenic effects of ATGL were mediated by DAG+FFA. We reasoned that, if the pro-tumorigenic effects of ATGL were mediated by DAG+FFA, then the impaired pathogenicity of sh-ATGL cancer cells might be rescued by treatment with exogenous sources of DAG+FFA. To test this hypothesis, we treated sh-ATGL HCC cells with 16 μM DAG+FFA. According to growth curves and CCK-8 assay results, sh-ATGL significantly inhibited HCC cell growth and this effect was completely rescued by treatment with 16 μM DAG +FFA (Fig. 2c and Additional file 4: Figure S2D). Colony formation assay further confirmed this process (Fig. 2d and Additional file 4: Figure S2E and F).

To determine whether these results were reproducible in vivo, we constructed an orthotopic xenografts HCC model in nude mice. Consistent with the results from in vitro assays, larger HCC tumors were observed in Huh7 overexpressing tumors compared with Huh7 control tumors (Fig. 2e-g). There were more Ki-67 positive cells in Huh7 overexpressing tumors compared with Huh7 control tumors (Fig. 2h and Additional file 6: Figure S4A). To explore the role of DAG and FFA on tumor growth in vivo, we injected 20 mg /kg DAG+FFA intraperitoneally daily for 5 weeks. The impaired tumor growth rate of HCCLM3 sh-ATGL tumors was completely rescued in mice treated with DAG+FFA. Notably, treatment with DAG+FFA significantly promoted the growth of HCCLM3 sh-control tumors (Fig. 2i-k). Furthermore, the IHC results indicated that Ki-67 was significantly decreased in sh-ATGL tumors and this effect was completely rescued in mice treatment with DAG +FFA (Fig. 2l and Additional file 6: Figure S4B). In addition, levels of DAG and FFA in mice orthotopic HCC tissues were higher in the Huh7 overexpressing tumors than in the control tumors. In contrast, the silencing of ATGL expression had the opposite effects (Fig. 2m). In summary, these results indicated that ATGL promotes HCC cell growth by maintaining elevated levels of DAG and FFA in vitro and in vivo.

### LncRNA-*NEAT1* modulates ATGL expression in HCC cells and disrupts the lipolysis of hepatoma cells via ATGL in vitro

**Fig. 3:**
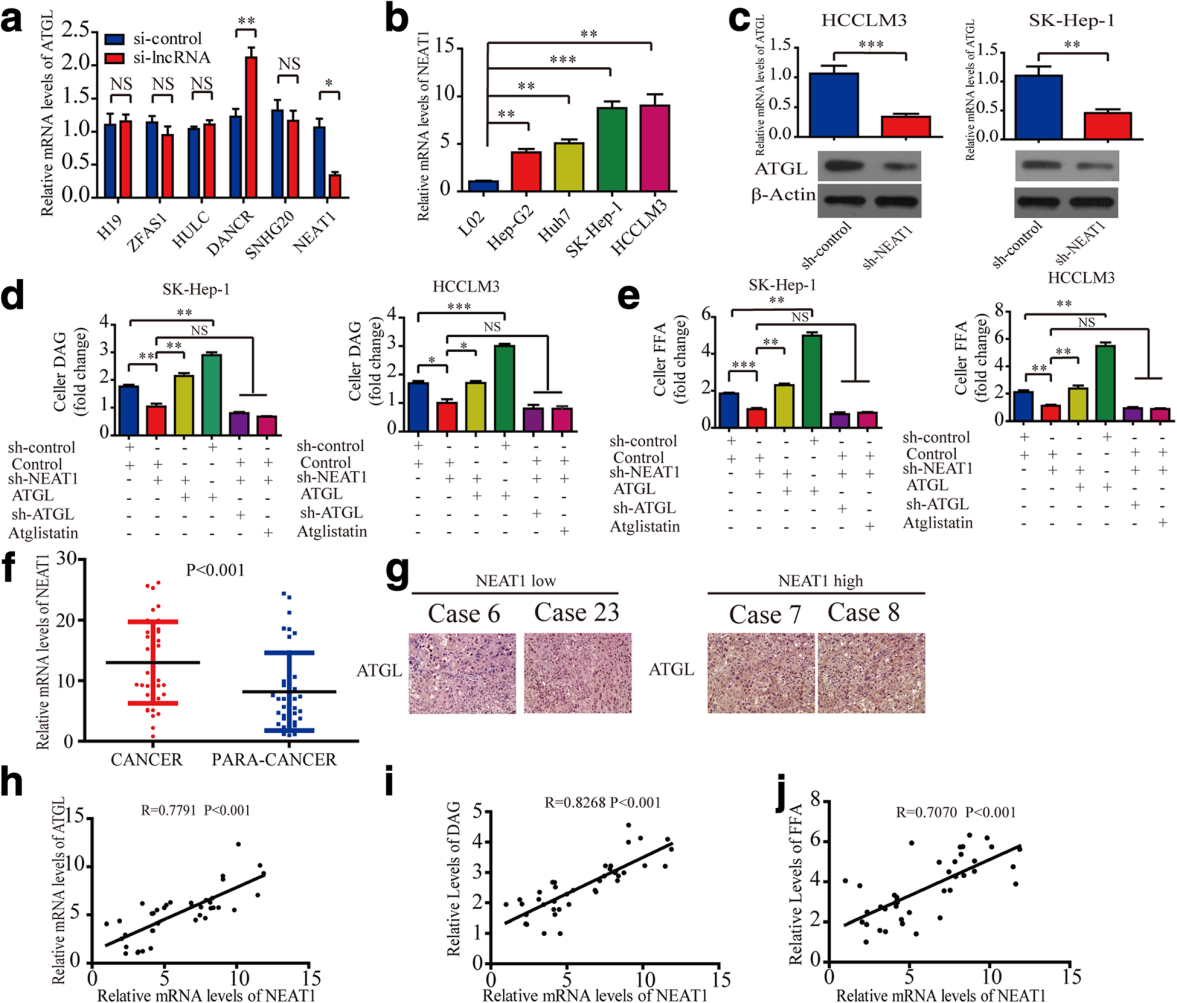
LncRNA-*NEAT1* modulates ATGL expression in HCC cells and disrupts the lipolysis of hepatoma cells via ATGL in vitro. **a** Effects of inhibition of *H19/ZFAS1/HULC/DANCR/SNHG20/NEAT1* expression by siRNA on ATGL expression as determined by real-time PCR analysis. **b** Real-time PCR analysis of *NEAT1* expression in HCC cell lines. **c** Real-time PCR analysis showing the effect of sh-*NEAT1* on ATGL expression in HCCLM3 and SK-Hep-1 cells (upper panel). Western blot analysis showing the effect of sh-*NEAT1* on ATGL expression in HCCLM3 and SK-Hep-1 cells (lower panel). **d** and **e** Intracellular DAG and FFA levels determine the effect of sh-*NEAT1* (or ATGL, sh-ATGL, Atglistatin) on lipolysis. **f** Real-time PCR analysis of *NEAT1* expression in 40 pairs of HCC cancer tissues and matched para-cancer tissues. **g** Representative images of IHC detection of ATGL in HCC tissues with low *NEAT1* expression (left panels) and high *NEAT1* expression (right panels). **h**, **i** and **j** Correlation analyses revealed that relative mRNA levels of *NEAT1* were positively associated with relative ATGL mRNA (or DAG, or FFA) levels in 40 HCC tissues. Pearson correlation coefficient was used to as a measure of association. Data are expressed as mean ± SD of three independent experiments. Statistical significance was concluded at *P < 0.05, **P < 0.01, ***P < 0.001; NS represents no statistical significance

Recent publications outline a regulatory role for LncRNA in lipid metabolism [18]. Thus, we speculated that lncRNAs might modulate ATGL expression. To test this hypothesis, we screened for and identified six lncRNAs co-expressed with ATGL in liver cancer tissues using the online software tool Co-LncRNA (http://www.bio-bigdata.com/Co-LncRNA/) [19]. Then, we knocked down the expression of these lncRNAs using siRNA to examine their effect on ATGL in HCC cells. The transfection efficiencies were detected by qRT-PCR (Additional file 7: Figure S5A). Our results demonstrated *DANCR* knockdown up-regulated the expression of ATGL and that *NEAT1* knockdown reduced the expression of ATGL in SK-Hep-1 cells (Fig. 3a). Given that resent studies has demonstrated that *DANCR* promotes HCC progression [20], and our study demonstrated that ATGL also promoted HCC cell growth. Therefore we pay attention to *NEAT1*. Next, we assessed *NEAT1* levels in HCC cells with different growth potentials and found that *NEAT1* RNA levels increased progressively from healthy liver cells to HCC cells with low growth potential and, finally, to HCC cells with high growth potential (Fig. 3b). Further studies revealed that knockdown of *NEAT1* by lentiviral transfection down-regulated ATGL mRNA and protein levels in SK-Hep-1 and HCCLM3 cells (Fig. 3c). The transfection efficiency was detected by qRT-PCR (Additional file 7: Figure S5B). However, we did not observe alterations in MAGL and HSL at mRNA or protein levels (Additional file 8: Figure S6.A and B).

Next, we investigated the effect of *NEAT1* on lipolysis in hepatoma cells. Reductions in intracellular FFA and DAG levels were observed following *NEAT1* knockdown, however overexpression of ATGL blocks this phenomenon. Simultaneously, knockdown of *NEAT1* with knockdown of ATGL (or treatment with Atglistatin) resulted in reduced DAG and FFA levels in HCCLM3 and SK-Hep-1 cells, however the effects were less than additive (Fig. 3d and e).

To confirm *NEAT1* was differentially expressed in HCC tissues, 40 pairs of HCC tissues and matching para-cancer tissues were used to evaluate *NEAT1* expression. As expected the expression of *NEAT1* was higher in HCC tissues than in the corresponding para-cancer liver tissues (Fig. 3f). Furthermore, IHC staining results indicated that ATGL was significantly higher in HCC tissues with relatively high *NEAT1* expression compared to that in tissues with relatively low *NEAT1* expression (Fig. 3g). Moreover, we found that *NEAT1* expression was positively correlated with tumor size. However, there was no correlation with HBV infection (Additional file 1: Table S2). In addition, *NEAT1* expression was found to be positively correlated with ATGL, DAG and FFA levels in HCC tissues (Fig. 3h-j). In summary, our results revealed that the *NEAT1* modulates ATGL expression in HCC cells and disrupts the lipolysis of hepatoma cells via ATGL.

### Abnormal lipolysis regulated by *NEAT1*-ATGL axis promotes HCC cell growth in vitro

**Fig. 4:**
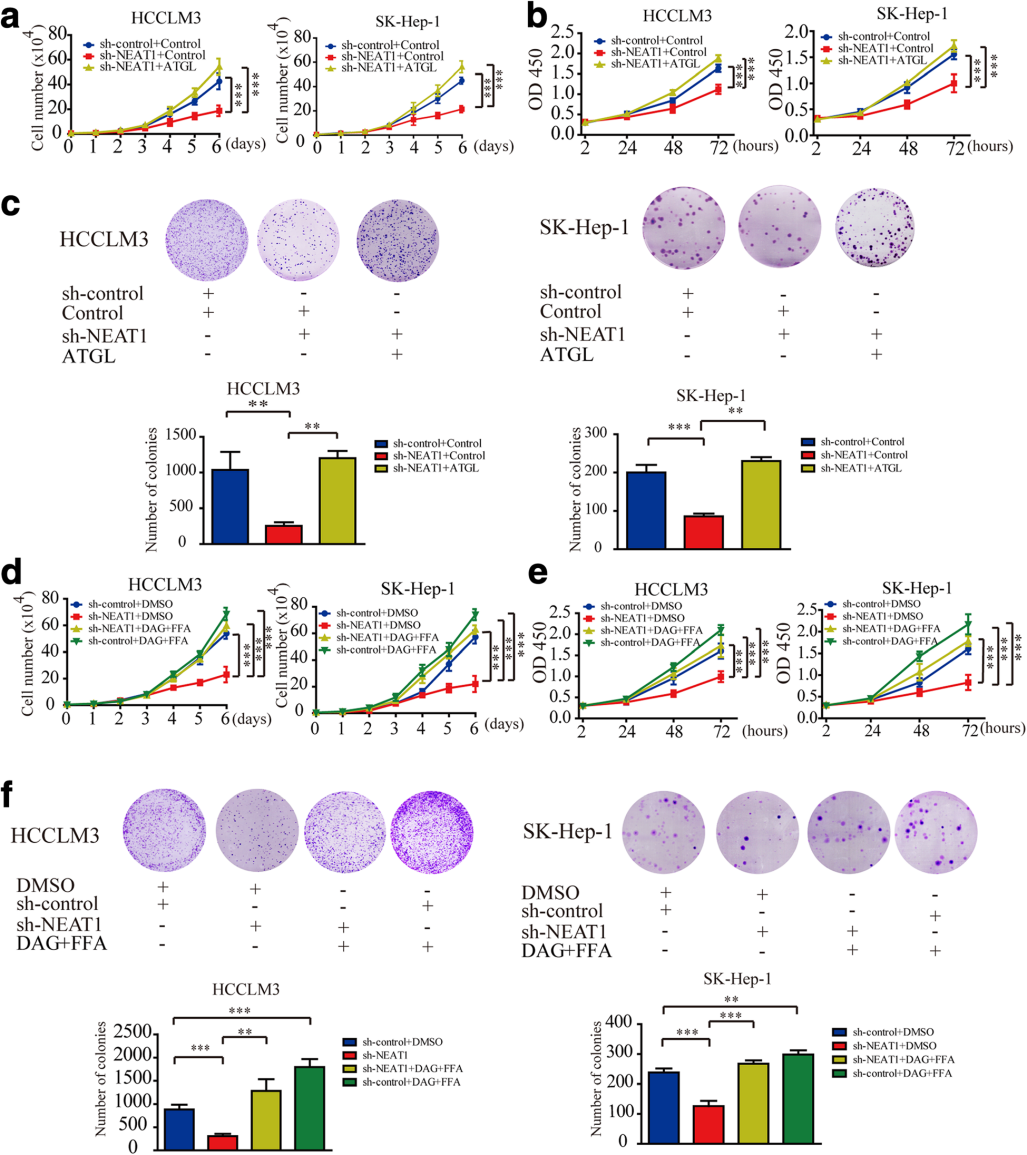
Abnormal lipolysis regulated by *NEAT1*-ATGL axis promotes HCC cell growth in vitro. **a** Growth curves for the indicated HCC cells were evaluated by the Trypan blue dye exclusion method. **b** CCK-8 assays showed that down-regulation of *NEAT1* expression inhibited the growth of HCCLM3 and SK-Hep-1 cells and this effect was rescued by up- regulation of ATGL. **c** Representative images of the cloning formation assay showed that down-regulation of *NEAT1* expression inhibited the growth of HCCLM3 and SK-Hep-1 cells and this effect was rescued by up-regulation of ATGL (upper panels). Number of colonies from three experiments were measured, and the results are presented as a bar graph (lower panels). **d** Growth curves for the indicated HCC cells were evaluated by the Trypan blue dye exclusion method. **e** CCK-8 assay showed that down-regulation of *NEAT1* expression inhibited the growth of HCCLM3 and SK-Hep-1 cells and this effect was completely rescued by treatment with 16 μM DAG+FFA. **f** Representative images of the cloning formation assay showed that down-regulation of *NEAT1* expression inhibited the growth of HCCLM3 and SK-Hep-1 cells and this effect was completely rescued by treatment with 16 μM DAG+FFA (upper panels). Number of colonies from three experiments were measured, and the results are presented as a bar graph(lower panels). Two-way ANOVA with the Bonferroni’s correction was used in different timepoints statistical analysis. Data are expressed as mean ± SD of three independent experiments. Statistical significance was concluded at **P < 0.01, ***P < 0.001

Next we evaluated the role of *NEAT1* in HCC. According to growth curves and CCK-8 assay results, we found that *NEAT1* knockdown significantly inhibited the proliferation of HCC cells. This inhibitory effect was reversed by the overexpression of ATGL (Fig. 4a and b). Colony formation assay further confirmed this effect (Fig. 4c). Given that recent publications indicated that *NEAT1* is transcriptionally regulated by the tumor suppressor p53(TP53) [21, 22], the survival effect of *NEAT1* may alter across different cancers. We also tested the TP53 gene mutations in 40 HCC tissues by PCR and direct sequencing. Given that exons 2-11 are coding exons of the p53 gene, we analyzed the sequence of p53 exons 2–11 for mutations. P53 mutations were found in 35% HCC tissues(14/40). The p53 mutations were located in exons 4-9 (Additional file 2: Table S6). Consistent with the published reports, our results showed that the expression of *NEAT1* was higher in TP53 wild-type tissues than in the TP-53 mutant liver tissues (Additional file 9: Figure S7A). Further, treatment with 10 μM Nutlin-3a(TP53 stabilizer) for 24 h resulted in higher *NEAT1* levels in TP53-wild-type Hep-G2 and SK-hep-1 cells but not in TP53 mutant Huh7 (p.Tyr220Cys) and HCCLM3 (p.Arg249Ser) cells (Additional file 9: Figure S7B). These results indicated that *NEAT1* is a TP53 target gene in HCC. Given that a recent publication reported that *NEAT1* expression can in turn prevent accumulation of TP53 [21]. We tested TP53 expression in *NEAT1* knockdown cells. Consistent with the previous report, TP53 was upregulated following *NEAT1* knockdown (Additional file 9: Figure S7C). To confirm this phenomenon, we also evaluated the expression of p21 and Bax, the target gene of p53. The results indicated that p21 and Bax was upregulated following *NEAT1* knockdown in HCC cell (Additional file 9: Figure S7D). These results indicated that *NEAT1* is a TP53 target gene in HCC and plays an oncogenic role in HCC. Further, ATGL is responsible for *NEAT1*-mediated HCC cell growth in vitro.

Considering that the pro-tumorigenic effects of ATGL were mediated by DAG and FFA, we hypothesized that the effect of *NEAT1* on HCC cell growth was also mediated by DAG and FFA. We found that *NEAT1* knockdown significantly inhibited the proliferation of HCC cells. This inhibitory effect was reversed by treatment with 16 μM DAG+FFA. Notably, treatment with DAG+FFA significantly promoted HCC cell growth (Fig. 4d-f).

Thus, we concluded ATGL and its products, DAG and FFA, are responsible for *NEAT1*-mediated HCC cell growth. Abnormal lipolysis regulated by *NEAT1*-ATGL axis promotes HCC cell growth in vitro.

### *NEAT1*-regulated abnormal lipolysis facilitates HCC cell growth in vivo

**Fig. 5:**
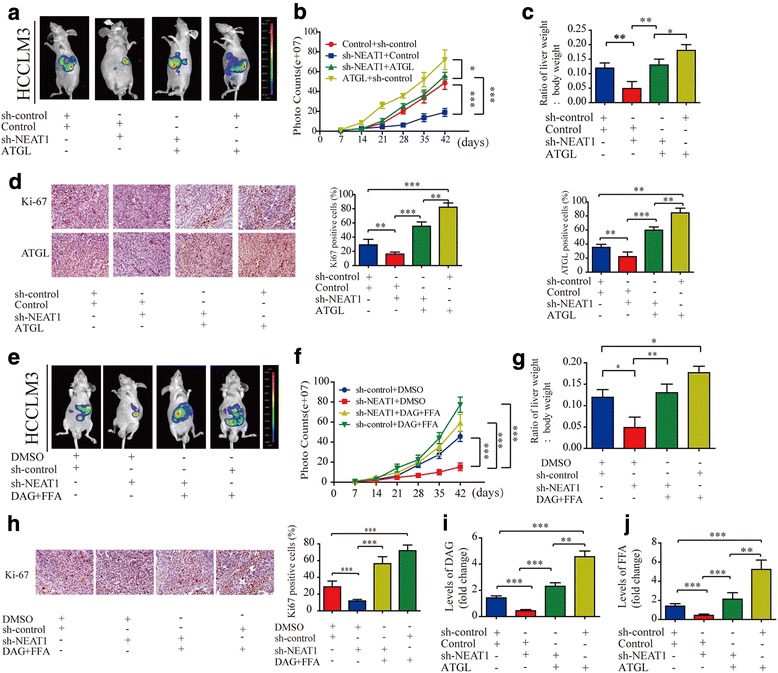
*NEAT1*-regulated abnormal lipolysis facilitates HCC cell growth in vivo. **a** Representative bioluminescence imaging of orthotopic HCCLM3 tumors. **b** Volume of HCCLM3 orthotopic tumors was determined at different timepoints. **c** The ratio of liver weight/body weight. **d** Representative images of immunohistochemical detection of Ki-67 and ATGL in orthotopic HCC tissues. **e** Representative bioluminescence imaging of HCCLM3 orthotopic HCC tumors in mice intraperitoneally injected with 20 mg/kg DAG+ FFA daily for 5 weeks. **f** Volume of HCCLM3 orthotopic tumors was determined at different timepoints. **g** The ratio of liver weight/body weight. **h** Representative images of immunohistochemical detection of Ki-67 in orthotopic HCC tissues. **i** and **j** Relative levels of DAG and FFA was determined in 100 mg orthotopic HCC tissues. Two-way ANOVA with the Bonferroni’s correction was used in different timepoints statistical analysis. Data are expressed as mean ± SD of three independent experiments. Statistical significance was concluded at *P < 0.05, **P < 0.01, ***P < 0.001

To better understand the hepatocarcinogenesis role of *NEAT1*-regulated abnormal lipolysis, we established orthotopic xenografts HCC model in nude mice. The results revealed that the inhibition of *NEAT1* expression led to smaller HCC tumors than control tumors, but this effect was blocked by co-transfection of sh-*NEAT1* + ATGL. Additionally, sh-*NEAT1* + ATGL tumors were smaller compared with ATGL overexpression tumors (Fig. 5a-c). Further, the IHC results indicated that Ki-67 and ATGL was significantly decreased in sh-*NEAT1* group and this effect was rescued in mice co-transfection of sh-*NEAT1* + ATGL (Fig. 5d). We then determined whether increased DAG+FFA delivery could rectify the tumor growth defect observed in sh-*NEAT1* cells in vitro. Orthotopic xenografts mice were injected with 20 mg/kg DAG+FFA intraperitoneally daily for 5 weeks. The impaired tumor growth rate of sh-*NEAT1* tumors was completely rescued in mice injected with DAG+FFA. Notably, treatment with DAG+FFA significantly promoted HCCLM3 sh-control tumor growth (Fig. 5e-g). Ki-67 IHC staining further confirmed this effect (Fig. 5h). In addition, levels of DAG and FFA in mice with orthotopic HCC tissues were lower in the sh-*NEAT1* tumors than in the control tumors, however, this effect was reversed by co-transfection of sh-*NEAT1* + ATGL (Fig. 5i and j). Taken together, these results indicate that *NEAT1*-regulated abnormal lipolysis contributes to HCC growth, and that this process is mediated by ATGL and its product, DAG+FFA, in vivo.

### Knockdown of *NEAT1* down-regulates ATGL expression by upregulating miR-124-3p levels

**Fig. 6:**
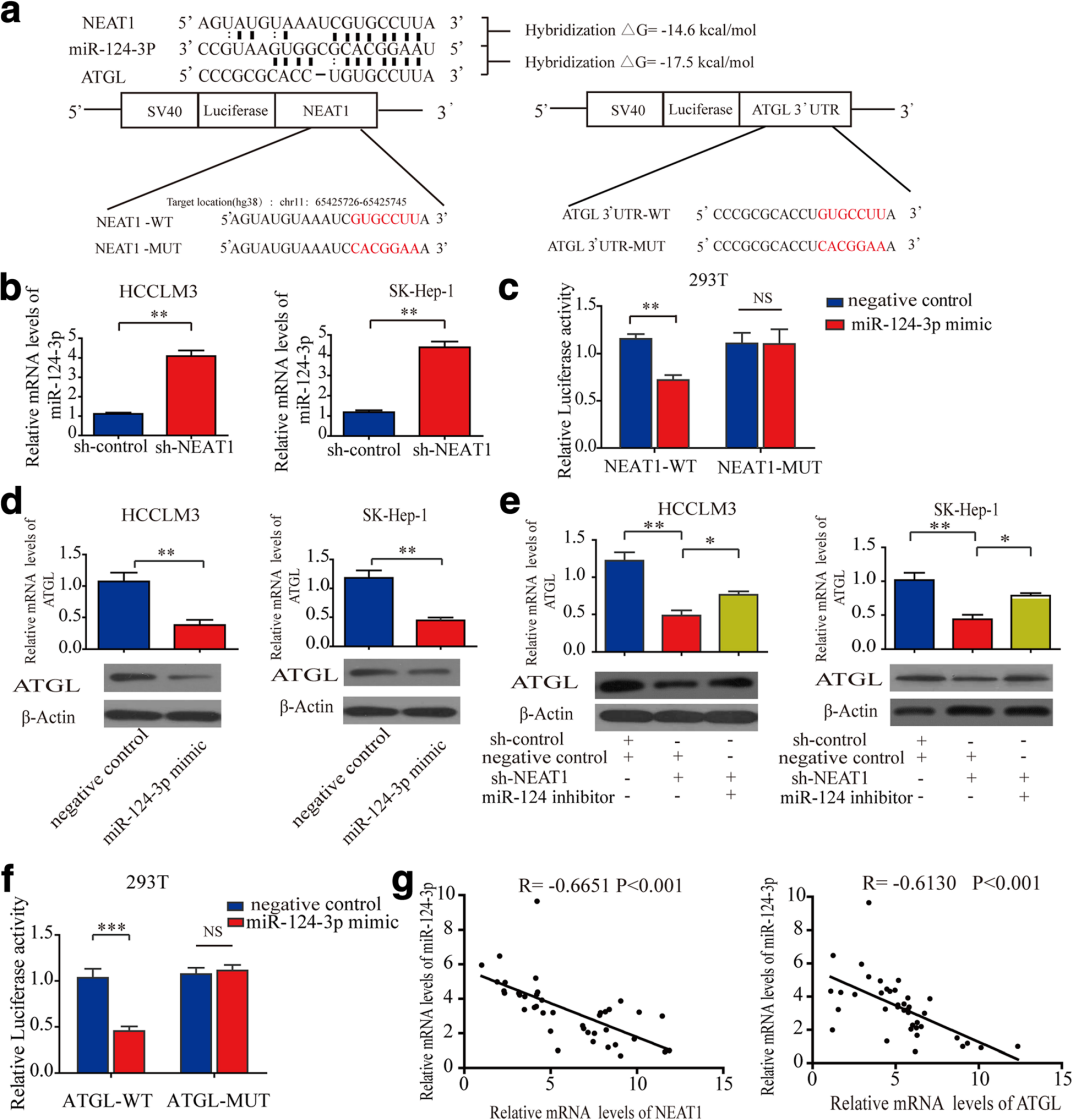
Knockdown of *NEAT1* down-regulates ATGL expression through upregulation of miR-124-3p levels. **a** Predicted conserved miR-124-3P binding sites of *NEAT1* and ATGL. The calculated free energy value of the hybrid is indicated. The generated mutant sites in *NEAT1* (or ATGL) 3′ UTR seed regions are indicated. **b** Real-time PCR analysis showing the effect of sh-*NEAT1* on miR-124-3p expression in HCCLM3 and SK-Hep-1 cells. **c** Dual-luciferase reporter assays revealed that co-transfection of *NEAT1-*WT with miR-124-3p in SK-Hep-1 cells decreased luciferase activity as compared with co-transfection using *NEAT1* muture. **d** Real-time PCR and western blot analysis showing the effect of up-regulation of miR-124-3p using mimic on ATGL expression in HCCLM3 and SK-Hep-1 cells. **e** Real-time PCR and Western blot results revealed that inhibition of *NEAT1* down-regulated ATGL expression, and this suppression was attenuated in cells with inhibited miR-124-3p. **f** Dual-luciferase reporter assays revealed that co-transfection of ATGL-WT with miR-124-3p in SK-Hep-1 cells decreased luciferase activity as compared to co-transfection using ATGL muture. **g** Correlation analyses revealed that expression levels of *NEAT1* (or ATGL) were inversely associated with those of miR-124-3p in 40 clinical HCC samples. Pearson correlation coefficient was used to as a measure of association. Data are expressed as mean ± SD of three independent experiments. Statistical significance was concluded at *P < 0.05, **P < 0.01, ***P < 0.001; NS represents no statistical significance

Several studies have confirmed that microRNAs are important target of lncRNAs [23, 24]. To identify whether any microRNAs represent potential targets that can be bind to ATGL and *NEAT1*, we used three online software tools (targetscan: http://www.targetscan.org/; microrna.org: http://www.microrna.org/; Starbase: http://starbase.sysu.edu.cn/.) to identify potential binding sites. We identified four microRNAs (hsa-miR-103a-3p, hsa-miR-214-3p, hsa-miR-124-3p and hsa-miR-107) that exhibited potential to bind to both *NEAT1* and ATGL. MiR-124-3p, which had the highest alignment score/Pct score is reported to have anti-cancer effects, therefore, we focused our investigation on this miRNA. The predictive information between miR-124-3p and the binding sites in the *NEAT1*/ATGL 3′-UTRs was illustrated in Fig. 6a. We further analysed the minimum free energy value of the hybrid between miR-124-3p and the binding site on the *NEAT1*/ATGL 3′-UTRs. The minimum free energy values were − 14.6 kcal/mol and − 17.5 kcal/mol, respectively, which are within the range of genuine miRNA-target pairs. Given the fact that miRNAs can target the nuclear lncRNA [25, 26], we first explored the subcellular distribution of miR-124-3p by FISH. The results showed that miR-124-3p was present both in the nucleus and cytoplasm (Additional file 10: Figure.S8A). Next we explored that whether *NEAT1* is able to regulate miR-124-3p expression. The results indicated that *NEAT1* knockdown led to a significant increase in miR-124-3p expression in HCC cells as assayed by qRT-PCR (Fig. 6b). In addition, a dual-luciferase reporter assay demonstrated that miR-124-3p mimics decreased the luciferase activities of NEAT1-WT but failed to influence the mutant, suggesting that miR-124-3p is able to directly bind to the NEAT1-WT target sites in 293 T cells (Fig. 6c). These results prove that NEAT1 regulate miR-124-3p expression in a sequence-specific and binding-dependent manner. Next, we overexpressed miR-124-3p using a miR-124-3p mimic. Western blot and qRT-PCR results indicated that exogenous miR-124-3p significantly reduced ATGL expression in HCCLM3 and SK-Hep-1 cells (Fig. 6d). Our previous results demonstrated that knockdown of *NEAT1* down-regulated ATGL expression, and we found that this suppression can be attenuated in HCC cells by inhibiting miR-124-3p (Fig. 6e). The transfection efficiencies of miR-124-3p were detected by qRT-PCR (Additional file 11: Figure S9A). In addition, a dual-luciferase reporter assay demonstrated that exogenous miR-124-3p reduced the luciferase activity of ATGL-WT but did not affect the luciferase activity of ATGL-MUT in 293 T cells (Fig. 6f). To further prove that miR-124-3p is involved in the cross-regulation between *NEAT1* and ATGL, We performed a rescue experiment with a dual-luciferase reporter assay using ATGL with mutant miR-124-3p binding sites. The results showed that depletion of *NEAT1* in 293 T cells inhibited the luciferase activity of ATGL-WT but not ATGL-MUT. Further, inhibition of miR-124-3p reversed this decrease in luciferase activity for ATGL-WT, but not for ATGL-MUT (Additional file 12: Figure S10A). Correlation analyses revealed that expression levels of *NEAT1* and ATGL were inversely associated with that of miR-124-3p in 40 clinical HCC samples (Fig. 6g). These results support the idea that *NEAT1* regulates ATGL expression through miR-124-3p.

To confirm the effect of miR-124-3p on lipolysis, we overexpressed miR-124-3p using miR-124-3p mimics in HCC cell lines. Our results indicated that both DAG and FFA levels were decreased in HCC cells (Additional file 13: Figure S11A and B). We also evaluated *NEAT1* and miR-124-3p expression in five pairs of HCC and matched non-tumor tissues by qRT-PCR. NEAT1 was upregulated and miR-124-3p was downregulated in the five HCC tissues compared with the matched non-tumor tissues (Additional file 14: Figure S12A).

### Knockdown of *NEAT1* attenuates HCC cell growth through miR-124-3p/ ATGL/ DAG+FFA/ PPARα signaling

**Fig. 7:**
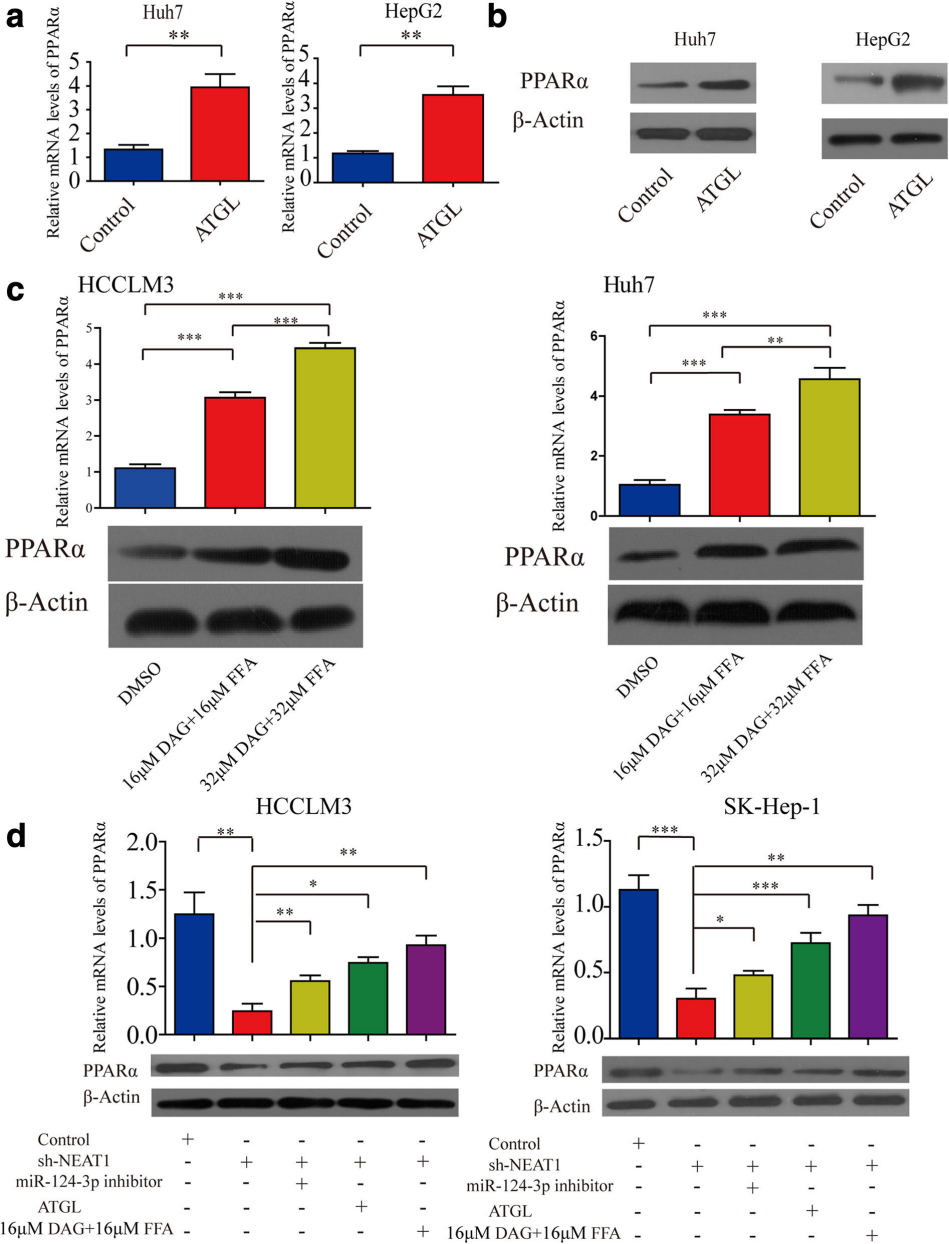
Knockdown of *NEAT1* attenuates HCC cell growth through miR-124-3p/ ATGL/ DAG+FFA/ PPARα signaling. **a** Real-time PCR analysis showing the effect of up-regulation of ATGL on PPARα expression in Huh7 and HepG2 cells. **b** Western blot analysis showing the effect of up-regulation of ATGL on PPARα expression in Huh7 and HepG2 cells. **c** Real-time PCR and western blot showing the effect of treatment with 16 μM and 32 μM DAG+FFA on PPARα expression in Huh7 and HCCLM3 cells. **d** Real-time PCR analysis and western blot analysis revealed that the sh-*NEAT1* down-regulated PPARα expression in HCCLM3 and SK-Hep-1 cells, whereas miR-124-3p inhibitor treatment (or overexpression of ATGL/treatment with DAG+FFA) blocked this process. Data are expressed as mean ± SD of three independent experiments. Statistical significance was concluded at *P < 0.05, **P < 0.01, ***P < 0.001

Considering that cancer cells have been reported to increase fatty acid oxidation (FAO) for cell survival due to compromised glucose uptake and ATGL-PPARα signaling have been reported to mediate FAO [27, 28], we hypothesized that *NEAT1* might serve as a means to mediate FAO through PPARα. To test this hypothesis, we first tested whether ATGL mediated PPARα in HCC cells. Our results showed that overexpression of ATGL significantly up-regulated PPARα expression in HCC cells (Fig. 7a and b). As various studies using a variety of biochemical techniques have firmly corroborated the direct physical association between fatty acids and PPARα and have thus established fatty acids as bona fide PPARα ligands [29], we hypothesized that DAG or FFA may active PPARα expression. To examine whether DAG+FFA regulates PPARα expression, we treated HCC cells with 16 μM and 32 μM DAG+FFA for 36 h. The resulting data demonstrated that DAG + FFA up-regulates the expression of PPARα in Huh7 and HCCLM3 cells in a dose dependent manner (Fig. 7c). In addition*,* our results showed that knockdown of *NEAT1* reduced PPARα levels, however, treatment with miR-124-3p inhibitor (or overexpression of ATGL/treatment with 16 μM DAG+FFA) blocked this process (Fig. 7d). The transfection efficiencies of miR-124-3p were detected by qRT-PCR (Additional file 11: Figure S9B). PPARα is a known oncogene in HCC [30]. Thus, we concluded that *NEAT1* mediates HCC cell growth via miR-124-3p/ATGL/DAG+FFA/PPARα signaling. Moreover, *NEAT1* may mediate FAO via PPARα, though this effect requires further study in the future.

### Combination of *NEAT1* and ATGL exhibits improved prognostic accuracy for HCC

**Fig. 8:**
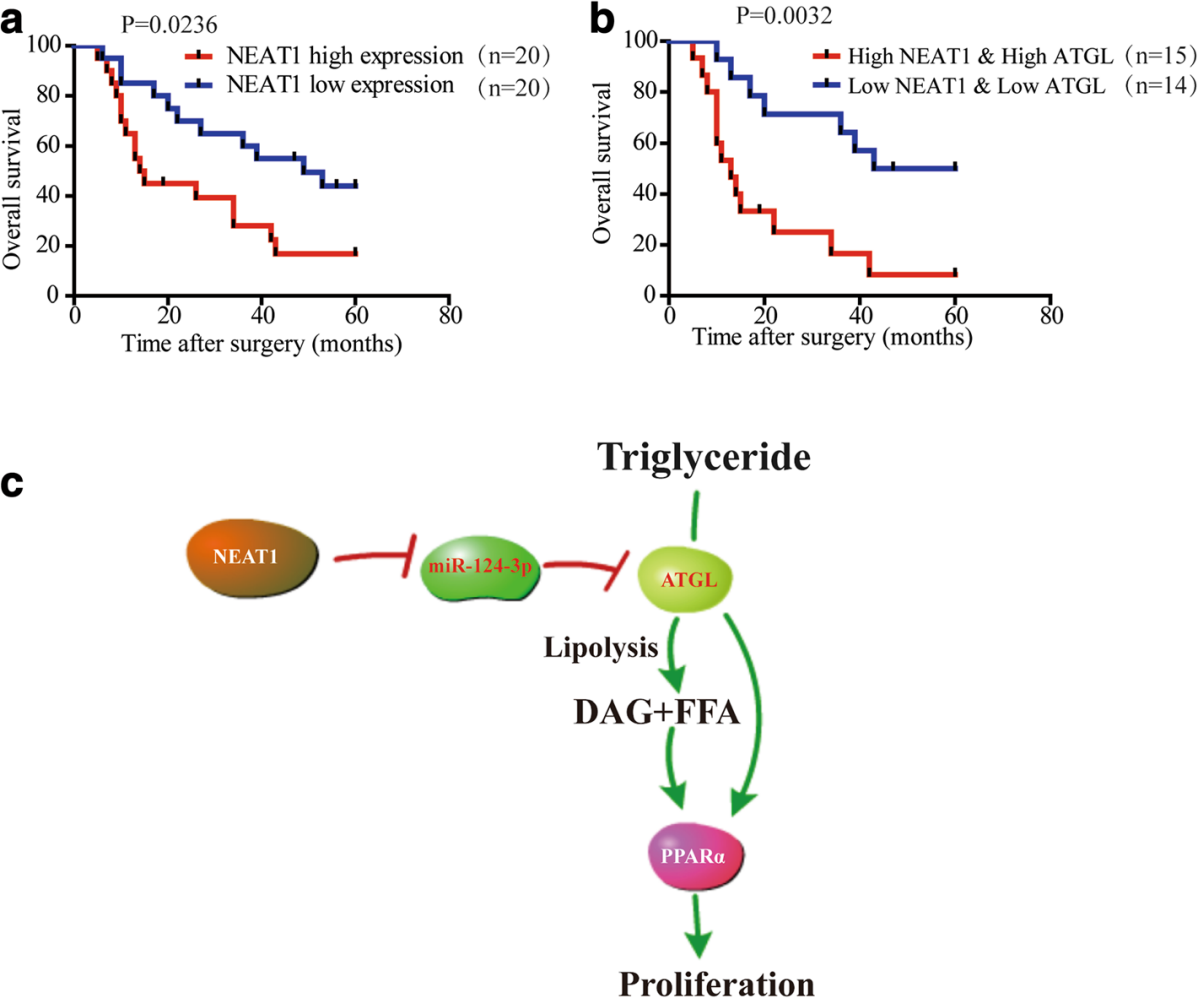
Combination of *NEAT1* and ATGL exhibits improved prognostic accuracy for HCC. **a** Kaplan-Meier analysis of overall survival in 40 patients indicated that high expression of *NEAT1* predicts poor prognosis. The cutoff lines to divide into high and low group was median value. **b** Kaplan-Meier analysis of overall survival in 40 patients indicated that patients with tumors exhibiting high levels of *NEAT1* and high levels of ATGL had lower survival. The cutoff lines to divide into high and low group was median value. **c** Schematic presentation of the mechanism underlying *NEAT1*-regulated abnormal lipolysis in hepatocarcinogenesis. *NEAT1* regulates ATGL expression via directly bind to miR-124-3p. *NEAT1* can influence the HCC proliferation through participating in lipolysis via ATGL. ATGL and its products, DAG and FFA, are responsible for *NEAT1*-mediated HCC cell growth through PPARα activation

Our results indicated *NEAT1* expression was negatively correlated with patient survival (Fig. 8a). It was previously shown that a combination of molecular markers can improve the prediction of patient prognosis [31]. We investigated whether this was the case for *NEAT1* and ATGL in HCC patients. Indeed, patients with tumors exhibiting high levels of *NEAT1* and high levels of ATGL had lower survival (Fig. 8b). Thus, combining *NEAT1* and *ATGL* expression improves the prediction of patient outcome.

## Discussion

In this study, we demonstrated that the *NEAT1*/miR-124-3p/ATGL pathway plays an important role in regulating abnormal lipolysis in HCC. In addition, *NEAT1*-mediated abnormal lipolysis facilitates HCC cell growth in vivo and in vitro.

Recent studies have demonstrated that fatty acids(FAs) may contribute to cancer progression through multiple mechanisms. Given that cancer cells can acquire FAs for their growth and proliferation through lipogenesis and lipolysis [4], we speculated that lipolysis may play a vital role in HCC development. Our results indicated that the lipolytic enzyme ATGL is highly expressed in HCC tissues and predicts poor prognosis. Although present studies have revealed that inhibition of ATGL attenuated the growth and motility of tumor cells [8], our study answered an important question: whether ATGL mediates lipolytic metabolic are responsible for this process. Our experimental results indicate that the pro-tumorigenic effects of ATGL were mediated by its DAG and FFA products. In addition, our results serve to explain, at least in part, high levels of DAG and FFA present in HCC tissues.

Recent publications outline a regulatory role for LncRNA in lipid metabolism [32], however, whether IncRNAs can influence the development of cancer through participating in lilpolytic metabolic, and the biochemical pathways involved in this process are both unknown. For this, we identified lncRNAs that are co-expressed with ATGL through the online tool Co-lncRNA. Based on this analysis, our results demonstrated that the down-regulation of *NEAT1* expression attenuates ATGL expression in HCC cells. In addition, we showed that *NEAT1* expression was positively correlated with ATGL levels in HCC tissues. These results indicated that *NEAT1* modulates ATGL expression in HCC. Further, reductions in intracellular FFA and DAG levels were observed following *NEAT1* knockdown. Our results demonstrated that *NEAT1* disrupts the lipolysis of hepatoma cells via ATGL. Notably, we determined that ATGL and its products, DAG and FFA, are responsible for *NEAT1* mediated HCC cell growth. These results demonstrated that *NEAT1*-modulated abnormal lipolysis promotes HCC cell growth in vivo and in vitro, providing new insight into the mechanism of HCC development.

Recent studies have been demonstrated that *NEAT1* is important in Adipogenesis [33]. Our results indicated that *NEAT1* also mediates lipolysis in HCC cells, indicating that *NEAT1* may be a central regulator in lipid metabolism. Other genes play similar central roles in lipid metabolism, including those encoding mTOR, PPARs (peroxisome proliferator-activated receptors), TNF-alpha and SIRT1 [34–37]. Further studies are needed to identify the effect of crosstalk between *NEAT1* and these genes.

*NEAT1* is up-regulated in various types of cancers and several studies have indicated multiple mechanisms of *NEAT1* up-regulation. First, the activation of hypoxia pathways is a feature of HCC. Previous studies indicated that *NEAT1* expression is upregulated by hypoxia through HIF-2α [38]. Second, *NEAT1* is also regulated by microRNAs. The findings suggest that the microRNA-*NEAT*1 regulatory network plays significant cellular and physiological roles, and that its dysregulation contributes to tumorigenesis [39]. Third, Some transcription factors such as Oct4 and estrogen receptor alpha have been reported to regulate *NEAT1* expression by directly binding *NEAT1* promoters [40, 41]. However, *NEAT1* is down-regulated and plays a tumor-suppressor role in specific cancer types, because NEAT1 is a target gene of wild type p53 [22, 42–44].. These seemingly contradictory results may reflect cell type-specific roles for *NEAT1* in tumorigenesis. An important mechanism that can explain this discrepancy is that *NEAT1* can in turn prevent accumulation of TP53 [21]. Our result confirmed this phenomenon in HCC cell lines. NEAT1 inhibits wild type p53 tumor suppressive functions through this negative feedback loop. However, whether this negative feedback loop is a universal phenomenon in human cancers needs further study. Additionally, P53 is the most commonly mutated gene in human cancers. According to the literature, NEAT1 is also highly expressed in the mutant p53 cell lines, such as lung cancer cell line: H1299; breast cancer cell line: MDA-MB-231; pancreatic cancer cell line PACN-1, colon cancer cell line: SW480 [12, 45–47]. This indicated some other NEAT1 regulator may play an important role in NEAT1 expression. Studies have revealed that some tumor-promoters, such as HIF-2, Oct4, and estrogen receptor alpha, can strongly enhance *NEAT1* expression [38, 40, 41]. NEAT1 could participate in the related gene pathway mentioned above to strongly promote tumor development.

Several studies have indicated that microRNAs can be important targets of lncRNAs [23, 24]. In this study, our results indicated that the interaction of *NEAT1* with ATGL might occupy the binding site of miRNAs so that suppression of ATGL by miR-124-3p would be significantly retarded. Meanwhile, dual-luciferase reporter assays demonstrated that both *NEAT1* and ATGL directly bind to miR-124-3p. We speculate that there may be a novel regulatory transcript-mediated release of ATGL from miRNA repression which would add to the known crosstalk within the established pathway.

To elucidate the mechanism of *NEAT1*-modulated abnormal lipolysis in hepatocarcinogenesis, we examined the expression of PPARα, the downstream target of ATGL [48]. PPARα is expressed mainly in the liver, heart, and muscles. It is a major regulator of fatty acid transport, catabolism, and energy homeostasis [49]. PPARα plays an important role in HCC proliferation [26]. Our results showed that ATGL expression enhances PPARα levels in HCC cells. Interestingly, our results also showed that treatment with DAG + FFA up-regulates the expression of PPARα in Huh7 and HCCLM3 cells in a dose dependent manner. Importantly, the knockdown of *NEAT1* down-regulated PPARα expression, but this process could be blocked by treatment with miR-124-3p inhibitor (or overexpression of ATGL/treatment with DAG+FFA). Therefore, we conclude that *NEAT1* promotes HCC cell growth through miR-124-3p/ATGL/DAG+FFA/PPARα signaling.

## Conclusions

In summary, we find that the lncRNA-*NEAT1* disrupts HCC cell lipolysis through ATGL. *NEAT1* regulates ATGL expression via competitively binding to miR-124-3p. Our results explain the high levels of DAG and FFA present in HCC tissues. ATGL and its products, DAG and FFA, are responsible for *NEAT1*-mediated HCC cell growth. Additionally, *NEAT1* mediates HCC cell growth through the miR-124-3p/ATGL/DAG+FFA/PPARα pathway. Importantly, Combination of *NEAT1* and ATGL exhibits improved prognostic accuracy for HCC. Thus, we here demonstrate that *NEAT1*-modulated abnormal lipolysis promotes HCC cell growth.

## Additional files

Additional file 1: **Table S1.** Relationship between ATGL expression and clinicopathological features of HCC patients. **Table S2.** Relationship between *NEAT1* expression and clinicopathological features of HCC patients. (DOCX 20 kb)

Additional file 2: **Table S3.** Sequences of siRNAs used in this study. **Table S4.** Sequences of primers used in this study. **Table S5.** Primers design of the tp53 gene. Table S6. Tp53 mutational analysis by PCR and direct sequencing. (DOCX 20 kb)

Additional file 3: **Figure S1.** ATGL mRNA was aberrantly expressed in HCC tissues and mediates lipolysis in HCC cells. A. Real-time PCR analysis of HSL, MAGL and ATGL expression in five pairs of HCC and matched non-tumor tissues. B. Transfection efficiency of ATGL and sh-ATGL as detected by western blot. C. Overexpression of ATGL increased intracellular FFA and DAG levels in Huh7 and HepG2 cell lines. D. ATGL knockdown (or treatment with Atglistatin) reduced intracellular FFA and DAG levels in HCCLM3 and SK-Hep-1 cell lines. Data are expressed as mean ± SD of three independent experiments. Statistical significance was concluded at **P < 0.01, ***P < 0.001. (TIF 436 kb)

Additional file 4: **Figure S2.** ATGL promotes HCC cell growth in vitro. A. Growth curves for the indicated HCC cells were evaluated by the Trypan blue dye exclusion method (left panel). CCK-8 assays showed that overexpression of ATGL promoted the growth of HepG2 cells (right panel). B. Representative images of the cloning formation assay showed that overexpression of ATGL promoted the growth of HepG2 cells. C. Number of colonies from three experiments were measured, and the results are presented as a bar graph. D. Growth curves for the indicated HCC cells were evaluated by the Trypan blue dye exclusion method (left panel). CCK-8 assays showed that ATGL knockdown inhibited the growth of SK-Hep-1 cells (right panel), however, this effect was completely rescued by treatment with 16 μM DAG+FFA. E. Representative images of the cloning formation assay showed that ATGL knockdown inhibited the growth of SK-Hep-1 cells, however, this effect was completely rescued by treatment with 16 μM DAG+FFA. F. Number of colonies from three experiments were measured, and the results are presented as a bar graph. Data are expressed as mean ± SD of three independent experiments. Statistical significance was concluded at *P < 0.05, **P < 0.01, ***P < 0.001. (TIF 614 kb)

Additional file 5: **Figure S3.** Treatment with DAG and FFA promote HCC cell growth. A. CCK-8 assays determined the effect of treatment with DAG at the concentrations of 8 μM, 16 μM, and 32 μM on HCC cell growth. B. CCK-8 assays determined the effect of treatment with FFA at the concentrations of 8 μM, 16 μM, and 32 μM on HCC cell growth. C. CCK-8 assays determined the effect of treatment with DAG, FFA, or DAG+FFA at a concentration of 16 μM on HCC cell growth. Data are expressed as mean ± SD of three independent experiments. Statistical significance was concluded at *P < 0.05, **P < 0.01, ***P < 0.001. (TIF 986 kb)

Additional file 6: **Figure S4.** Ki-67 positive cells in IHC. A. More Ki-67 positive cells in Huh7 overexpressing tumors compared with Huh7 control tumors B. Ki-67 positive cells were decreased in sh-*NEAT1* tumors, however this effect was completely rescued in mice tumors injected DAG+FFA. Data are expressed as mean ± SD. Statistical significance was concluded at **P < 0.01, ***P < 0.001. (TIF 682 kb)

Additional file 7: **Figure S5.** Transfection efficiency as detected by qRT-PCR. A. Transfection efficiency of *H19/ZFAS1/HULC/DANCR/SNHG20/NEAT1* in SK-Hep-1 cells as detected by qRT-PCR. B. Transfection efficiency of *NEAT1* as detected by qRT-PCR. C. Transfection efficiency of sh-*NEAT1* and sh-ATGL in Fig. 3d, e as detected by western blot and qRT-PCR. Data are expressed as mean ± SD of three independent experiments. Statistical significance was concluded at ***P < 0.001. (TIF 343 kb)

Additional file 8: **Figure S6.** *NEAT1* does not mediate MAGL or HSL expression in HCC cells. A. Real-time PCR analysis determined the effects of sh-*NEAT1* on MAGL and HSL in HCC cells. B. Western blot analysis determined the effect of sh-*NEAT1* on MAGL and HSL in HCC cells. Data are expressed as mean ± SD of three independent experiments. NS represents no statistical significance. (TIF 588 kb)

Additional file 9: **Figure S7.** *NEAT1* is a TP53 target gene in HCC. A. The expression of *NEAT1* was higher in TP53 wild-type tissues (*n* = 26) than in the TP-53 mutant liver tissues (*n* = 14). B. Treatment with Nutlin-3a resulted in higher *NEAT1* levels in TP53 wild-type Hep-G2 and SK-hep-1 cells but not in TP53 mutant Huh7 and HCCLM3 cells. C. Western blot analysis determined TP53 was upregulated following *NEAT1* knockdown in SK-Hep-1 and Hep-G2 cells. D. Western blot analysis determined p21 and Bax was upregulated following *NEAT1* knockdown in SK-Hep-1 and Hep-G2 cells. Data are expressed as mean ± SD of three independent experiments. Statistical significance was concluded at *P < 0.05, **P < 0.01, NS represents no statistical significance. (TIF 530 kb)

Additional file 10: **Figure S8.** The subcelluar distribution of miR-124-3p was explored by FISH. A. Representative images showing localization of CY3-miR-124-3p in HCC cell lines. 18S, probe for 18S rRNA; U6, probe for U6 snRNA. (TIF 739 kb)

Additional file 11: **Figure S9.** The transfection efficiencies of miR-124-3p were detected by qRT-PCR. A. The mRNA levels of miR-124-3p in Fig. 6e as detected by qRT-PCR. B The mRNA levels of miR-124-3p in Fig. 7d as detected by qRT-PCR. Data are expressed as mean ± SD of three independent experiments. Statistical significance was concluded at ***P < 0.001. (TIF 820 kb)

Additional file 12: **Figure S10.** Dual-luciferase reporter assays reveals miR-124-3p is involved in the crossregulation between *NEAT1* and ATGL. A. Dual-luciferase reporter assays revealed that depletion of *NEAT1* in 293 T cells inhibited the luciferase activity of ATGL-WT but not ATGL-MUT. Further, inhibition of miR-124-3p reversed this decrease in luciferase activity for ATGL-WT, but not for ATGL-MUT. Data are expressed as mean ± SD. Statistical significance was concluded at **P < 0.01. NS represents no statistical significance. (TIF 685 kb)

Additional file 13: **Figure S11.** The effect of miR-124-3p on lipolysis. A. Treatment with miR-124-3p mimic decrease intracellular DAG levels in SK-hep-1 and HCCLM3 cells B. Treatment with miR-124-3p mimic decrease intracellular FFA levels in SK-hep-1 and HCCLM3 cells**.** Data are expressed as mean ± SD of three independent experiments. Statistical significance was concluded at **P < 0.01, ***P < 0.001. (TIF 832 kb)

Additional file 14: **Figure S12.** *NEAT1* and miR-124-3p mRNA was aberrantly expressed in 5 pairs of HCC and matched non-tumor tissues. A. Real-time PCR analysis of *NEAT1* and miR-124-3p expression in five pairs of HCC and matched non-tumor tissues. Data are expressed as mean ± SD of three independent experiments. (TIF 678 kb)

## Abbreviations

ATGL/PNPLA2: Adipose triglyceride lipase
DAG: Diacylglycerol
FAS: Fatty acid synthesis
FFA: Free fatty acid
HCC: Hepatocellular carcinoma
HSL: Hormone-sensitive lipase
IHC: Immunohistochemistry
MAG: Monoacylglycerol
MAGL: Monoglyceride lipase
*NEAT1*: Nuclear paraspeckle assembly transcript 1
PCR: Polymerase chain reaction
PPARα: Peroxisome proliferator activated receptor alpha
TAG: Triglyceride
