# Supplementary material for: Long non-coding RNA NEAT1-modulated abnormal lipolysis via ATGL drives hepatocellular carcinoma proliferation
Source: Mol Cancer. 2018 May 15;17:90. doi: 10.1186/s12943-018-0838-5 (PMC5953401; doi:10.1186/s12943-018-0838-5)
Supplement: Supplementary file 1 — Table S1. Relationship between ATGL expression and clinicopathological features of HCC patients. Table S2. Relationship between NEAT1 expression and clinicopathological features of HCC patients. (DOCX 20 kb) [file 12943_2018_838_MOESM1_ESM.docx]

| Features | Relative ATGL expression levels | | *P* Value |
| --- | --- | --- | --- |
|  | High (n=20) | ATGL (n=20) |  |
| Age |  |  |  |
| ≤50 | 4 | 3 | 0.667 |
| >50 | 16 | 17 |  |
| Gender |  |  |  |
| Male | 18 | 16 | 0.376 |
| Female | 2 | 4 |  |
| AFP (μg/L) |  |  |  |
| ≤20 | 5 | 7 | 0.490 |
| >20 | 15 | 13 |  |
| HBV |  |  |  |
| Positive | 16 | 13 | 0.288 |
| Negative | 4 | 7 |  |
| Liver cirrhosis |  |  |  |
| Yes | 14 | 17 | 0.256 |
| No | 6 | 3 |  |
| Tumor diameter(cm) |  |  |  |
| ≤5 | 13 | 5 | 0.011 |
| >5 | 7 | 15 |  |

Table S1. Relationship between ATGL Expression and Clinicopathologic Features of HCC Patients(n=40)

Note: HCC patients were divided into ATGL ‘High’ group (Relative fold change was higher than the median) and ‘Low’ group (Relative fold change was lower than the median).

Abbreviations: HBV Positive, hepatitis B virus surface antigen positive; AFP, alphafetoprotein.

Differences among variables were assessed by χ2 or Fisher’s exact χ2 test.

| Features | Relative NEAT1 expression levels | | *P* Value |
| --- | --- | --- | --- |
|  | High  (n=20) | Low  (n=20) |  |
| Age |  |  |  |
| ≤50 | 3 | 6 | 0.256 |
| >50 | 17 | 14 |  |
| Gender |  |  |  |
| Male | 15 | 16 | 0.705 |
| Female | 5 | 4 |  |
| AFP (μg/L) |  |  |  |
| ≤20 | 10 | 4 | 0.047 |
| >20 | 10 | 16 |  |
| HBV |  |  |  |
| Positive | 17 | 14 | 0.256 |
| Negative | 3 | 6 |  |
| Liver cirrhosis |  |  |  |
| Yes | 13 | 11 | 0.519 |
| No | 7 | 9 |  |
| Tumor diameter(cm) |  |  |  |
| ≤5 | 3 | 15 | <0.001 |
| >5 | 17 | 5 |  |

Table S2. Relationship between NEAT1 Expression and Clinicopathologic Features of HCC Patients(n=40)

Note: HCC patients were divided into NEAT1 ‘High’ group (Relative fold change was higher than the median) and ‘Low’ group (Relative fold change was lower than the median).

Abbreviations: HBV Positive, hepatitis B virus surface antigen positive; AFP, alphafetoprotein.

Differences among variables were assessed by χ2 or Fisher’s exact χ2 test.
