# Supplementary material for: Long non-coding RNA NEAT1-modulated abnormal lipolysis via ATGL drives hepatocellular carcinoma proliferation
Source: Mol Cancer. 2018 May 15;17:90. doi: 10.1186/s12943-018-0838-5 (PMC5953401; doi:10.1186/s12943-018-0838-5)
Supplement: Supplementary file 2 — Table S3. Sequences of siRNAs used in this study. Table S4. Sequences of primers used in this study. Table S5. Primers design of the tp53 gene. Table S6. Tp53 mutational analysis by PCR and direct sequencing. (DOCX 20 kb) [file 12943_2018_838_MOESM2_ESM.docx]

Table S3: Sequences of the siRNA used in this study

| LncRNA | Sequence |
| --- | --- |
| HULC | CCUCCAGAACUGUGAUCCAdTdT |
| SNHG20 | GCCUAGGAUCAUCCAGGUUTT |
| DANCR | AGCCAACTATCCCTT CAGT |
| NEAT1 | UGGUAAUGGUGGAGGAAGAUU |
| H19 | GCAGGACAUGACAUGGUCC |
| ZFAS1 | UCCAAAAUCCAUUCUGUACCC |
| control | UCCGCUGACGACAAGGAUG |

| miR-124-3p | Forward:CGGGCTAAGGCACGCGGT | Reverse:CAGCCACAAAAGAGCACAAT |
| --- | --- | --- |
| NEAT1 | Forward:TTTGTGCTTGGAACCTTGCT | Reverse: TCAACGCCCCAAGTTATTTC |
| ATGL/PNPLA2 | Forward:GTGTCAGACGGCGAGAATG | Reverse:TGGAGGGAGGGAGGGATG |
| PPARα | Forward:GGCGAGGATAGTTCTGGAAGC | Reverse:CACAGGATAAGTCACCGAGGAG |
| MAGL | Forward:ATGGTAGAGTTCGCGCCCTTGTTT | Reverse:TCATCATAACTCCAAGTGCTGGTCA |
| HSL | Forward:CTCAGTGTGCTCTCCAAGTG | Reverse: CACCCAGGCGGAAGTCTC |
| HULC | Forward:ATCTGCAAGCCAGGAAGAGTC | Reverse:CTTGCTTGATGCTTTGGTCTGT |
| SNHG20 | Forward:ATGGCTATAAATAGATACACGC | Reverse:GGTACAAACAGGGAGGGA |
| DANCR | Forward:GCGCCACTATGTAGCGGGTT | Reverse:TCAATGGCTTGTGCCTGTAGTT |
| H19 | Forward:TGCTGCACTTTACAACCACTG | Reverse: TGGTGTCTTTGATGTTGGGC |
| ZFAS1 | Forward:ACGTGCAGACATCTACAACCT | Reverse:TACTTCCAACACCCGCAT |
| U6 | Forward:GCTTCGGCAGCACATATACTAAAAT | Reverse:CGCTTCACGAATTTGCGTGTCAT |
| β-Actin | Forward:GCAAGCAGG AGTATGACGAG | Reverse:CAAATAAAGCCA TGCCAATC |

Table S4: Sequences of the primers used in this study

| Target exons | Primer | Sequences | Product length (bp) |
| --- | --- | --- | --- |
| Exon 2, 3 | Forward: | CTTGGGTTGTGGTGAAAC | 537 |
|  | Reverse: | GGGGACTGTAGATGGGTG |  |
| Exon 4 | Forward: | TGGTAAGGACAAGGGTT | 534 |
|  | Reverse: | CACATTAAGTGGGTAAACTAT |  |
| Exon 5, 6 | Forward: | AGTGACAGAGCAAGACCCTA | 677 |
|  | Reverse: | CAAATAAGCAGCAGGAGAAA |  |
| Exon 7 | Forward: | GCTGAGGAAGGAGAATGG | 465 |
|  | Reverse: | AAAGAAAACTGAGTGGGA |  |
| Exon 8, 9 | Forward: | GTGGTTGGGAGTAGATGG | 568 |
|  | Reverse: | GTTGGTGTTCTGAAGTTAGTTA |  |
| Exon 10 | Forward: | CCGTCATAAAGTCAAACAA | 384 |
|  | Reverse: | TGAGGCAAGAATGTGGTT |  |
| Exon 11 | Forward: | AGAGTGAAACTCCGTCTCAAA | 588 |
|  | Reverse: | TTACATCTCCCAAACATCCC |  |

Table S5. Primers design of the tp53 gene.

| Exon | Nucleotide  change | Amino acid  change |
| --- | --- | --- |
| Exon 4 | c.325T>G | p.Phe109Val |
| Exon 5 | c.419_432del14 | p.Thr140_Gln144del |
|  | c.476C>A | p.Ala159Asp |
|  | c.395A>C | p.Lys132Thr |
|  | c.451C>T | p.Pro151Ser |
| Exon 6 | c.659A>G | p.Tyr220Cys |
| Exon 7 | c.733G>A | p.Gly245Ser |
|  | c.743_747del5 | p.Arg249_Pro250del |
| Exon 8 | c.824_825delGT | p.cys276Leu fs |
|  | c.817C>T | p.Arg273Cys |
|  | c.892G>T | p.Glu298Ter |
|  | c.916C>T | p.Arg306Ter |
| Exon 9 | c.976G>T | p.Glu326Ter |
|  | c.976G>T | p.Glu326Ter |

Table S6. p53 mutational analysis by PCR and direct sequencing.
