# Supplementary figures and images for: Long non-coding RNA NEAT1-modulated abnormal lipolysis via ATGL drives hepatocellular carcinoma proliferation
Source: Mol Cancer. 2018 May 15;17:90. doi: 10.1186/s12943-018-0838-5 (PMC5953401; doi:10.1186/s12943-018-0838-5)

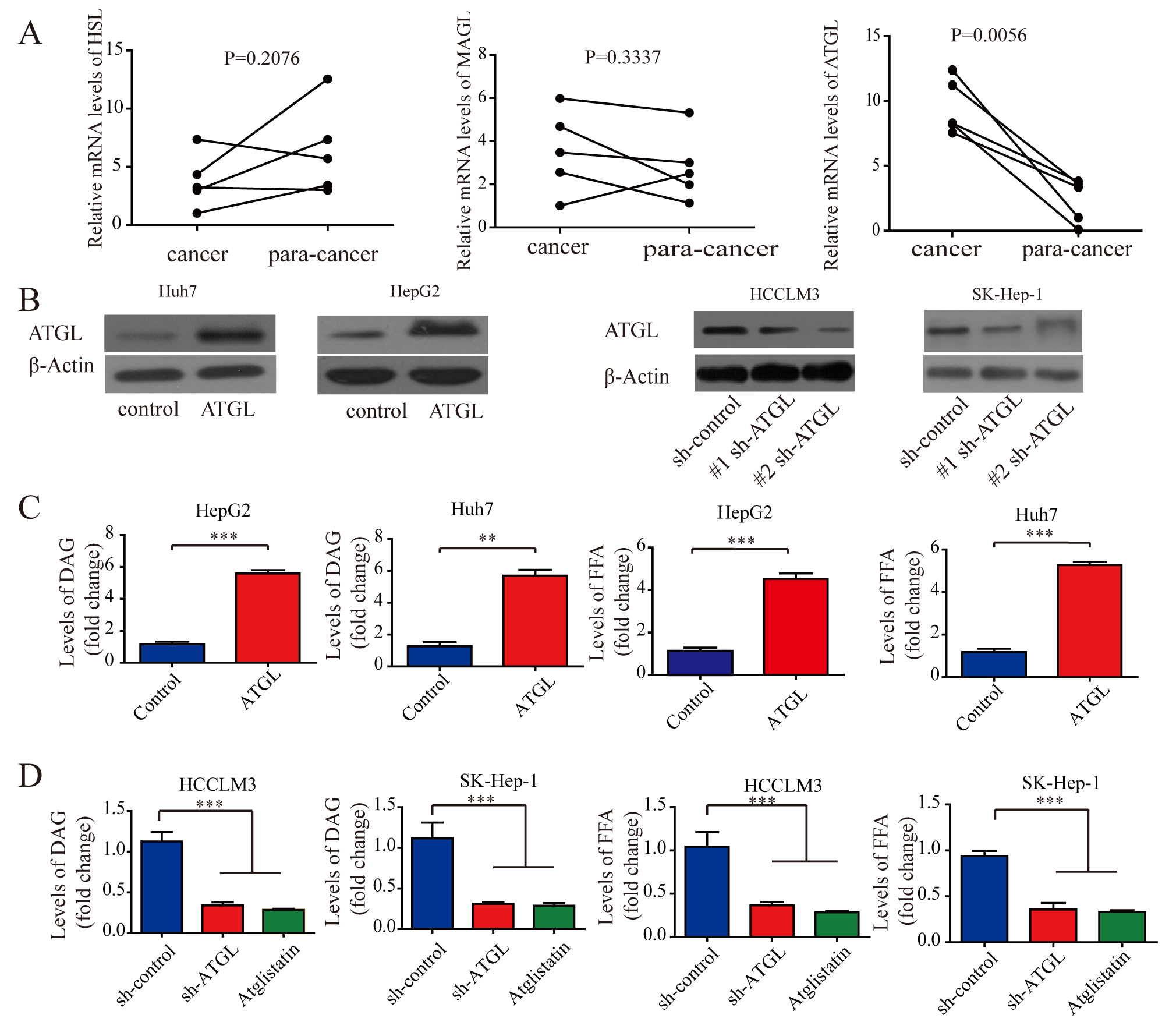

Supplement: Supplementary file 3 — Figure S1. ATGL mRNA was aberrantly expressed in HCC tissues and mediates lipolysis in HCC cells. A. Real-time PCR analysis of HSL, MAGL and ATGL expression in five pairs of HCC and matched non-tumor tissues. B. Transfection efficiency of ATGL and sh-ATGL as detected by western blot. C. Overexpression of ATGL increased intracellular FFA and DAG levels in Huh7 and HepG2 cell lines. D. ATGL knockdown (or treatment with Atglistatin) reduced intracellular FFA and DAG levels in HCCLM3 and SK-Hep-1 cell lines. Data are expressed as mean ± SD of three independent experiments. Statistical significance was concluded at **P < 0.01, ***P < 0.001. (TIF 436 kb) [file 12943_2018_838_MOESM3_ESM.tif]

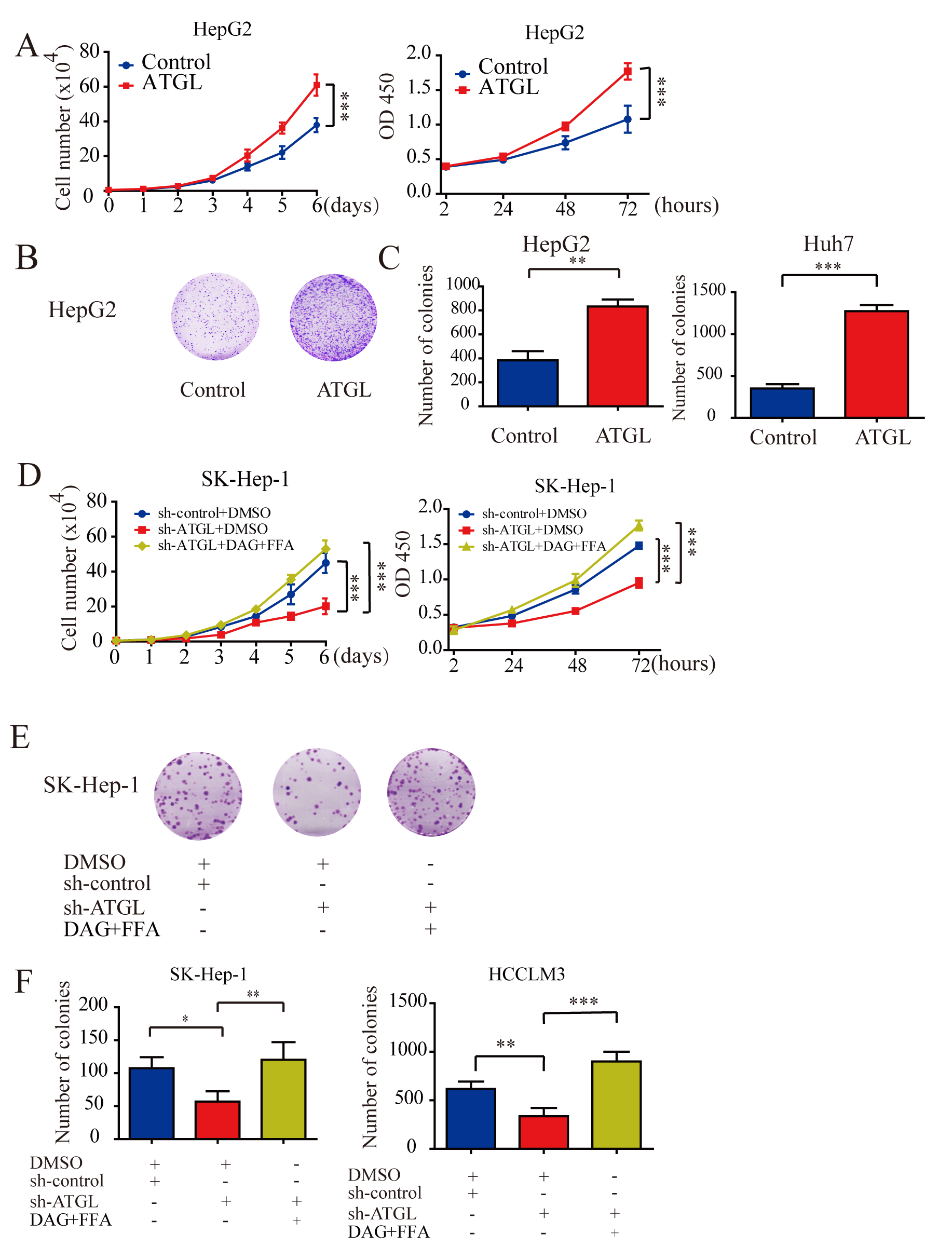

Supplement: Supplementary file 4 — Figure S2. ATGL promotes HCC cell growth in vitro. A. Growth curves for the indicated HCC cells were evaluated by the Trypan blue dye exclusion method (left panel). CCK-8 assays showed that overexpression of ATGL promoted the growth of HepG2 cells (right panel). B. Representative images of the cloning formation assay showed that overexpression of ATGL promoted the growth of HepG2 cells. C. Number of colonies from three experiments were measured, and the results are presented as a bar graph. D. Growth curves for the indicated HCC cells were evaluated by the Trypan blue dye exclusion method (left panel). CCK-8 assays showed that ATGL knockdown inhibited the growth of SK-Hep-1 cells (right panel), however, this effect was completely rescued by treatment with 16 μM DAG+FFA. E. Representative images of the cloning formation assay showed that ATGL knockdown inhibited the growth of SK-Hep-1 cells, however, this effect was completely rescued by treatment with 16 μM DAG+FFA. F. Number of colonies from three experiments were measured, and the results are presented as a bar graph. Data are expressed as mean ± SD of three independent experiments. Statistical significance was concluded at *P < 0.05, **P < 0.01, ***P < 0.001. (TIF 614 kb) [file 12943_2018_838_MOESM4_ESM.tif]

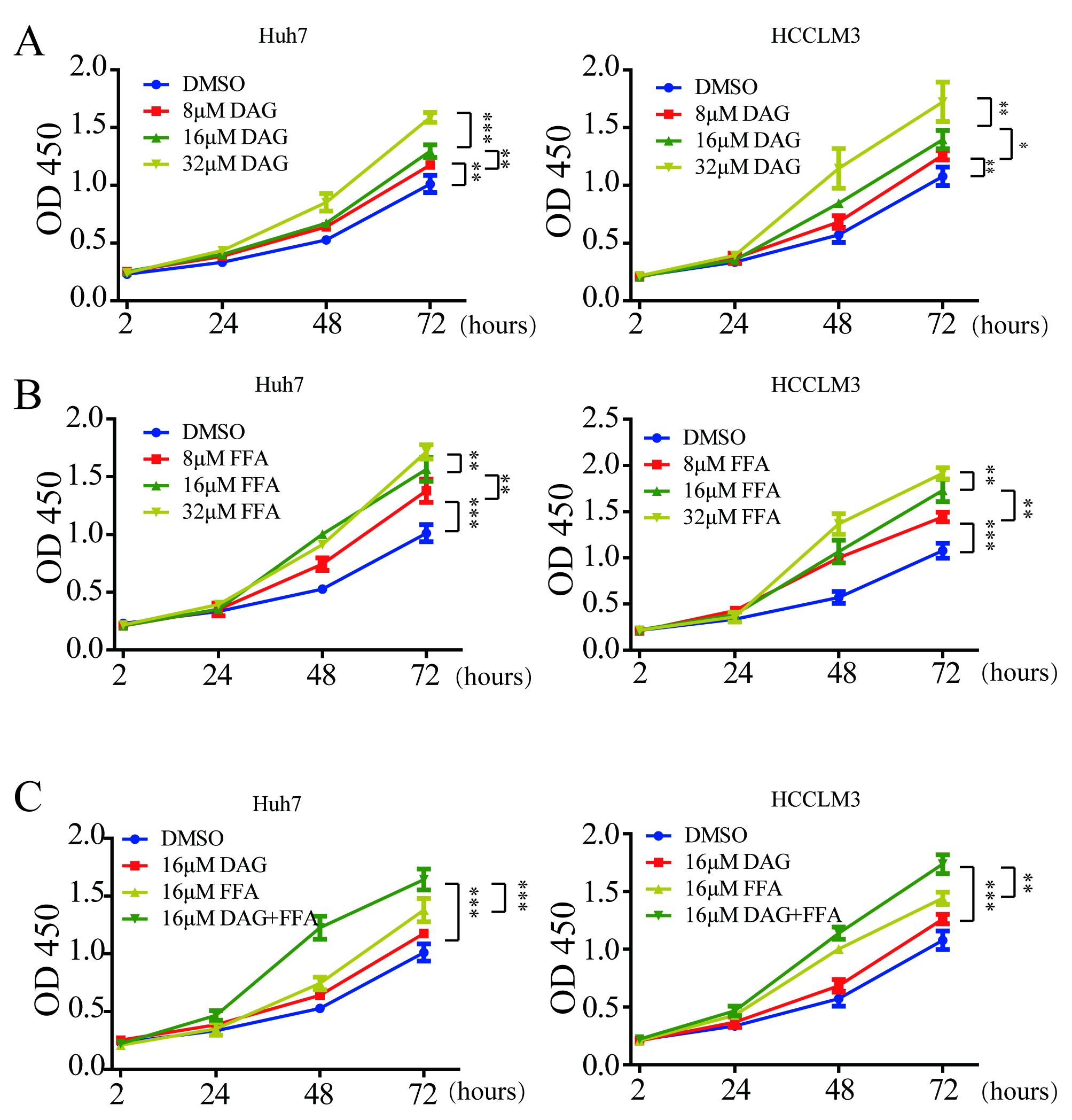

Supplement: Supplementary file 5 — Figure S3. Treatment with DAG and FFA promote HCC cell growth. A. CCK-8 assays determined the effect of treatment with DAG at the concentrations of 8 μM, 16 μM, and 32 μM on HCC cell growth. B. CCK-8 assays determined the effect of treatment with FFA at the concentrations of 8 μM, 16 μM, and 32 μM on HCC cell growth. C. CCK-8 assays determined the effect of treatment with DAG, FFA, or DAG+FFA at a concentration of 16 μM on HCC cell growth. Data are expressed as mean ± SD of three independent experiments. Statistical significance was concluded at *P < 0.05, **P < 0.01, ***P < 0.001. (TIF 986 kb) [file 12943_2018_838_MOESM5_ESM.tif]

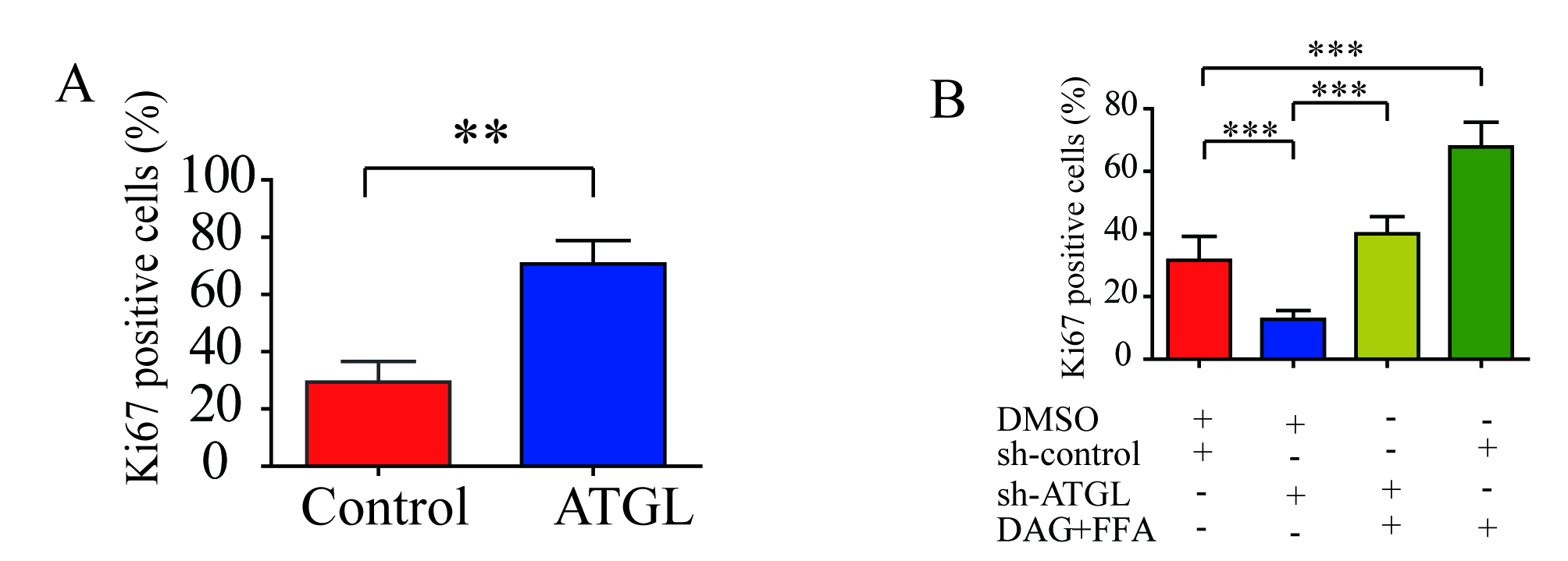

Supplement: Supplementary file 6 — Figure S4. Ki-67 positive cells in IHC. A. More Ki-67 positive cells in Huh7 overexpressing tumors compared with Huh7 control tumors B. Ki-67 positive cells were decreased in sh-NEAT1 tumors, however this effect was completely rescued in mice tumors injected DAG+FFA. Data are expressed as mean ± SD. Statistical significance was concluded at **P < 0.01, ***P < 0.001. (TIF 682 kb) [file 12943_2018_838_MOESM6_ESM.tif]

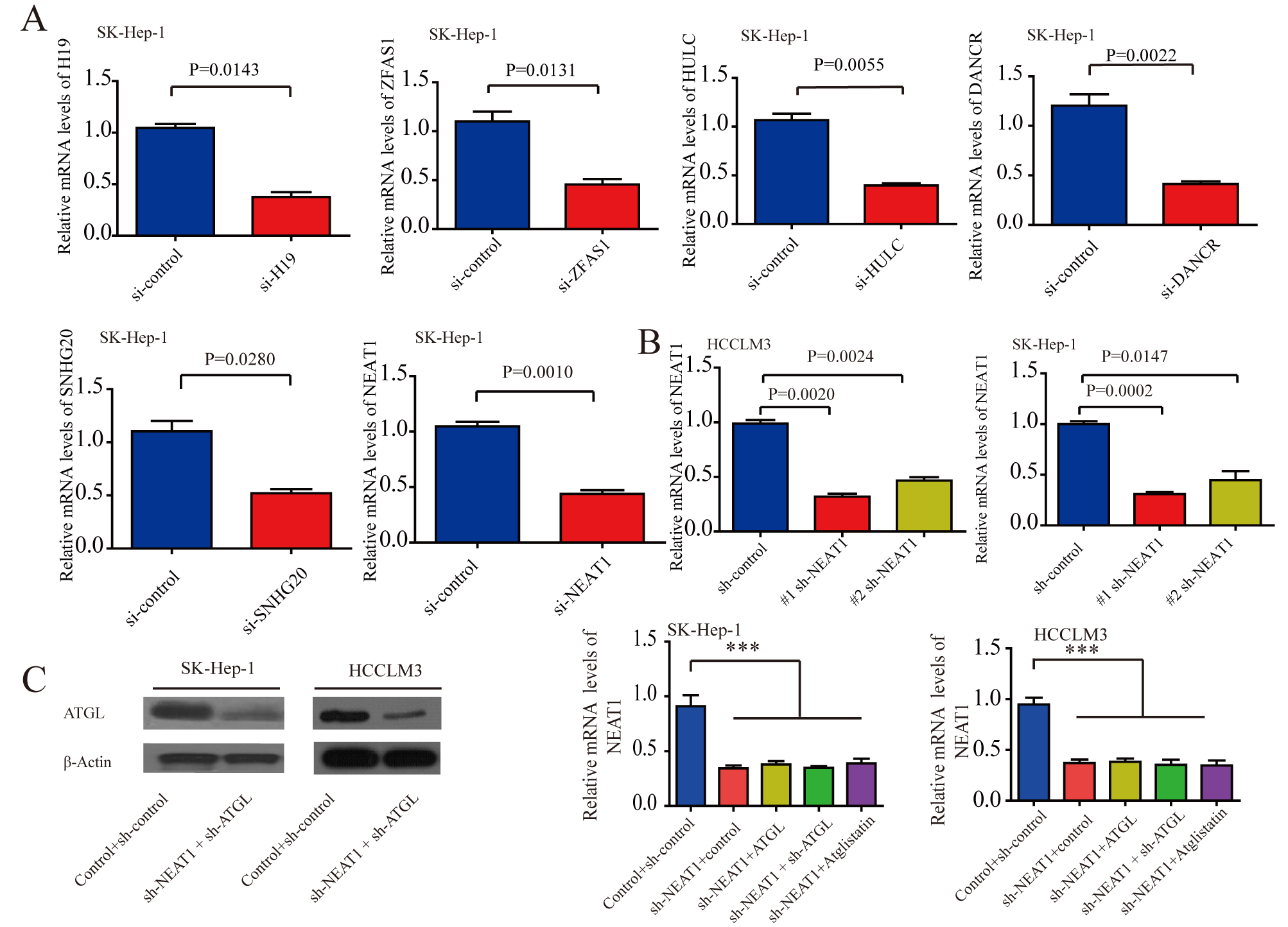

Supplement: Supplementary file 7 — Figure S5. Transfection efficiency as detected by qRT-PCR. A. Transfection efficiency of H19/ZFAS1/HULC/DANCR/SNHG20/NEAT1 in SK-Hep-1 cells as detected by qRT-PCR. B. Transfection efficiency of NEAT1 as detected by qRT-PCR. C. Transfection efficiency of sh-NEAT1 and sh-ATGL in Fig. 3d, e as detected by western blot and qRT-PCR. Data are expressed as mean ± SD of three independent experiments. Statistical significance was concluded at ***P < 0.001. (TIF 343 kb) [file 12943_2018_838_MOESM7_ESM.tif]

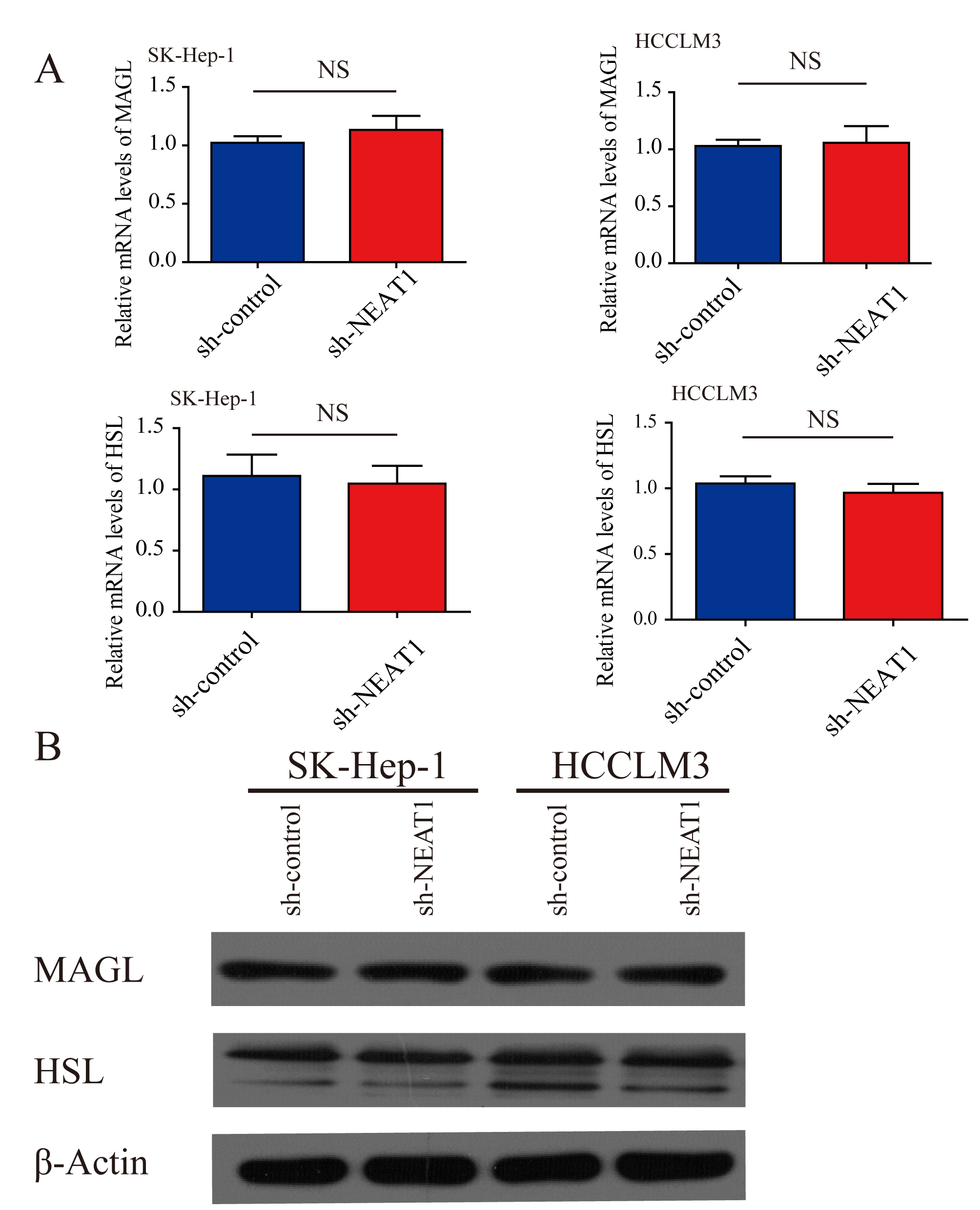

Supplement: Supplementary file 8 — Figure S6. NEAT1 does not mediate MAGL or HSL expression in HCC cells. A. Real-time PCR analysis determined the effects of sh-NEAT1 on MAGL and HSL in HCC cells. B. Western blot analysis determined the effect of sh-NEAT1 on MAGL and HSL in HCC cells. Data are expressed as mean ± SD of three independent experiments. NS represents no statistical significance. (TIF 588 kb) [file 12943_2018_838_MOESM8_ESM.tif]

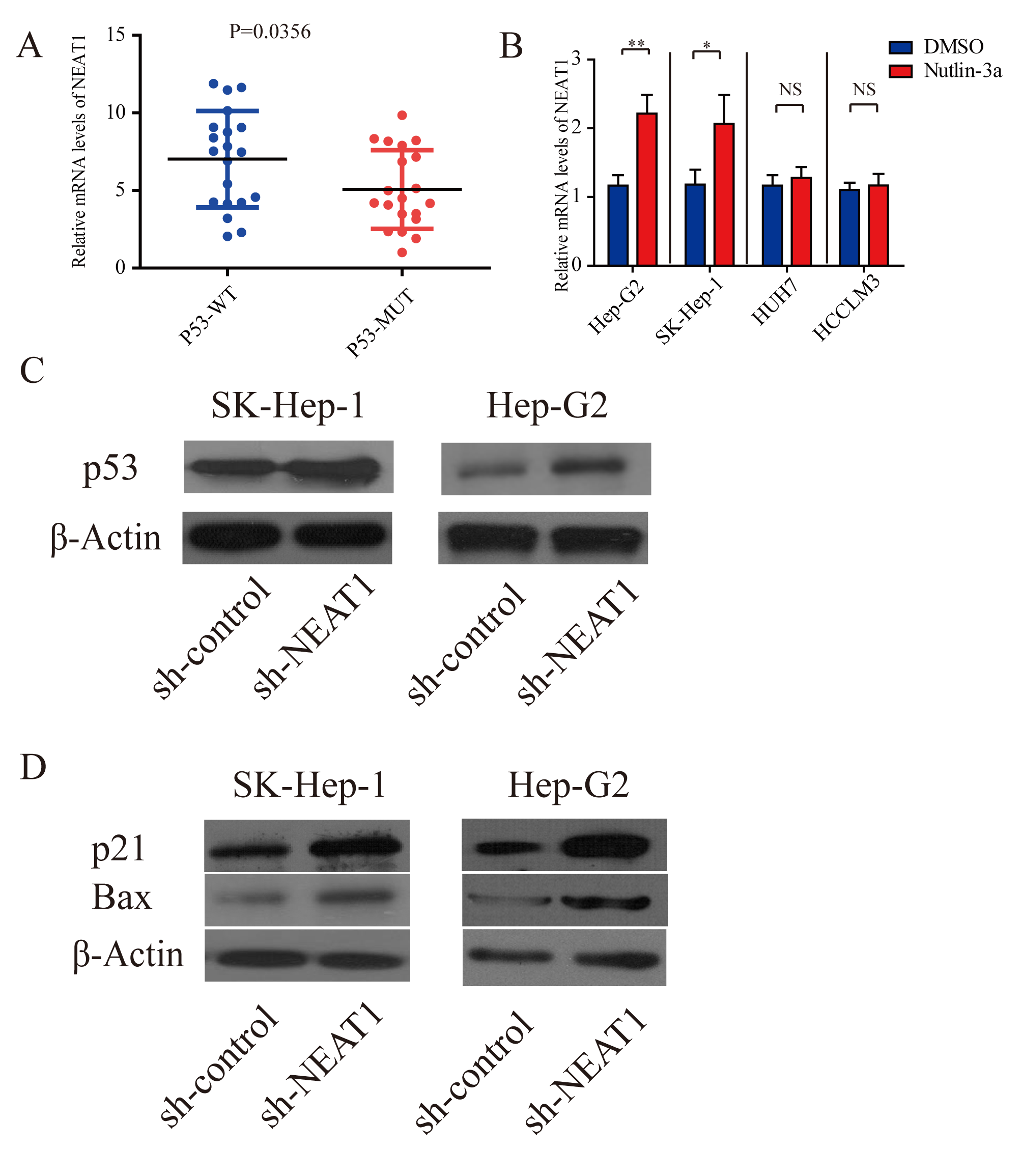

Supplement: Supplementary file 9 — Figure S7. NEAT1 is a TP53 target gene in HCC. A. The expression of NEAT1 was higher in TP53 wild-type tissues (n = 26) than in the TP-53 mutant liver tissues (n = 14). B. Treatment with Nutlin-3a resulted in higher NEAT1 levels in TP53 wild-type Hep-G2 and SK-hep-1 cells but not in TP53 mutant Huh7 and HCCLM3 cells. C. Western blot analysis determined TP53 was upregulated following NEAT1 knockdown in SK-Hep-1 and Hep-G2 cells. D. Western blot analysis determined p21 and Bax was upregulated following NEAT1 knockdown in SK-Hep-1 and Hep-G2 cells. Data are expressed as mean ± SD of three independent experiments. Statistical significance was concluded at *P < 0.05, **P < 0.01, NS represents no statistical significance. (TIF 530 kb) [file 12943_2018_838_MOESM9_ESM.tif]

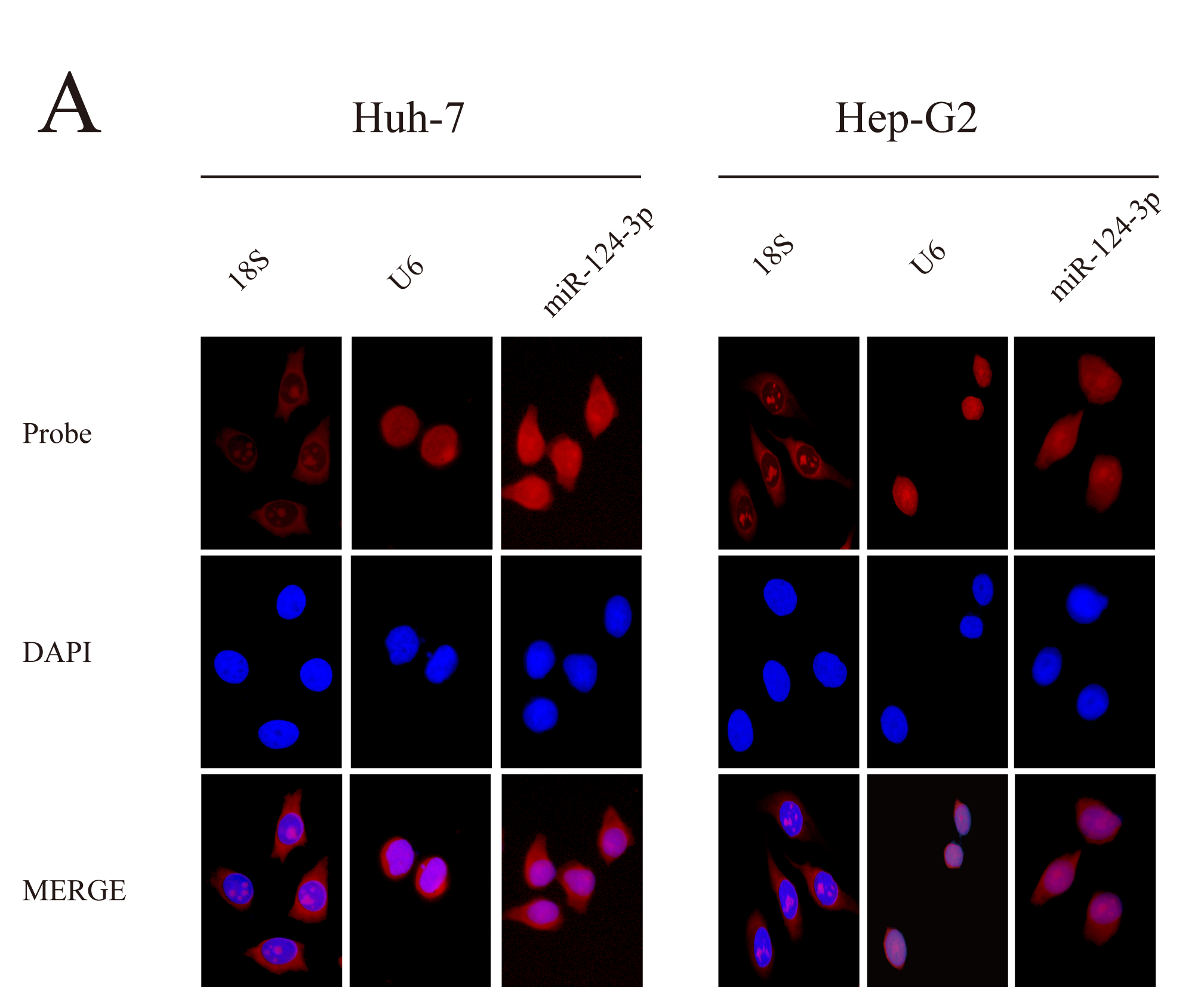

Supplement: Supplementary file 10 — Figure S8. The subcelluar distribution of miR-124-3p was explored by FISH. A. Representative images showing localization of CY3-miR-124-3p in HCC cell lines. 18S, probe for 18S rRNA; U6, probe for U6 snRNA. (TIF 739 kb) [file 12943_2018_838_MOESM10_ESM.tif]

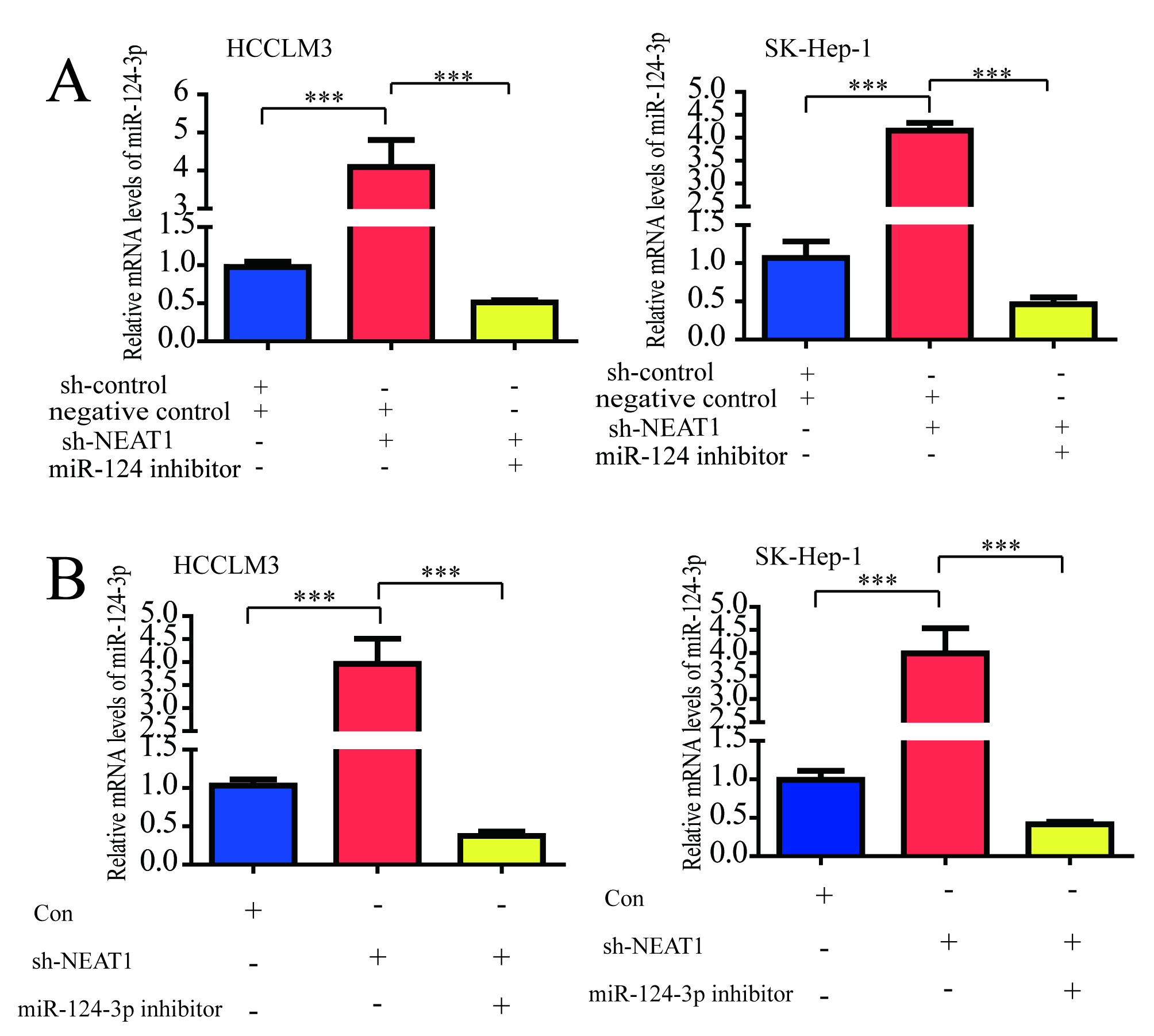

Supplement: Supplementary file 11 — Figure S9. The transfection efficiencies of miR-124-3p were detected by qRT-PCR. A. The mRNA levels of miR-124-3p in Fig. 6e as detected by qRT-PCR. B The mRNA levels of miR-124-3p in Fig. 7d as detected by qRT-PCR. Data are expressed as mean ± SD of three independent experiments. Statistical significance was concluded at ***P < 0.001. (TIF 820 kb) [file 12943_2018_838_MOESM11_ESM.tif]

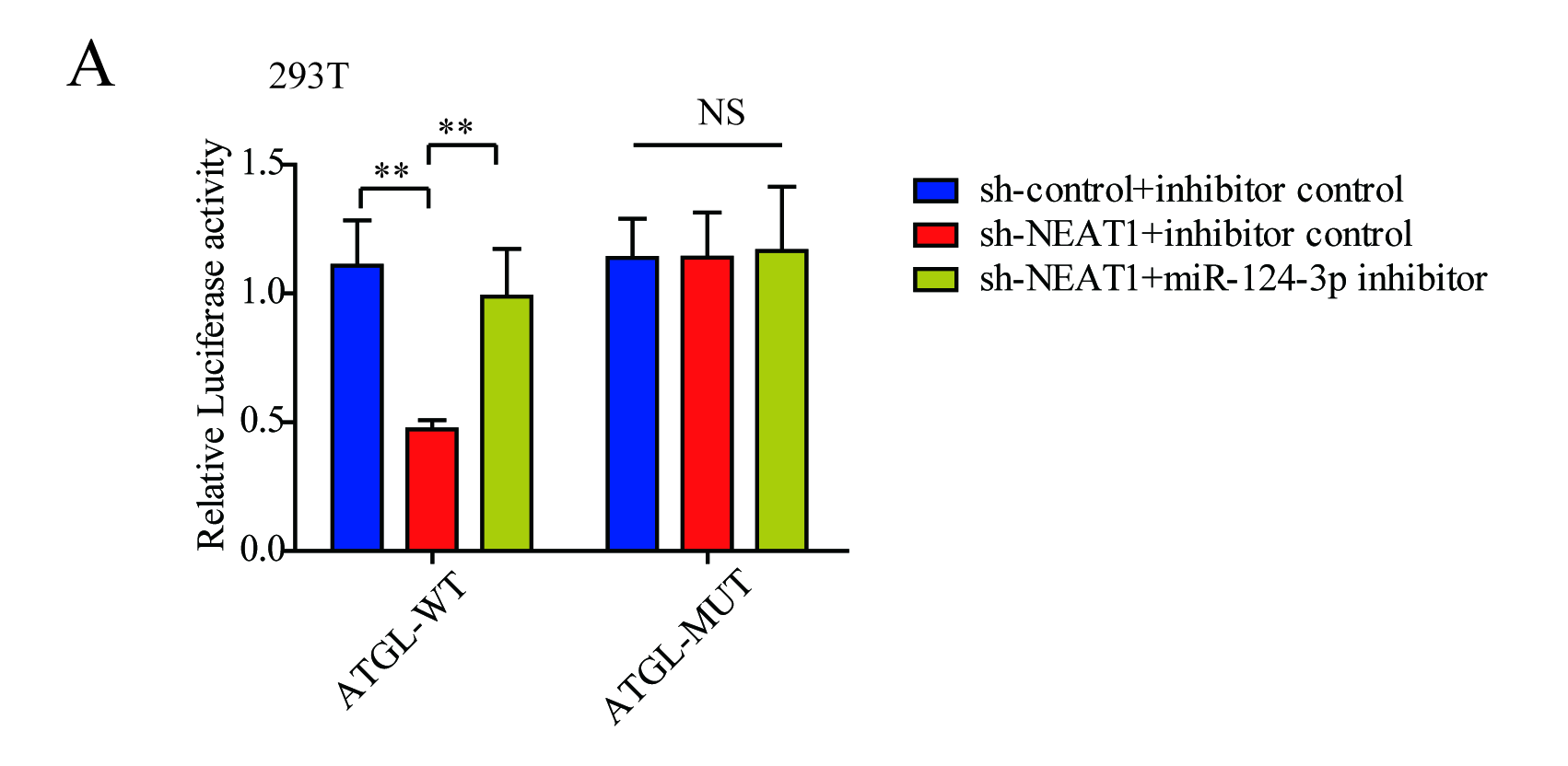

Supplement: Supplementary file 12 — Figure S10. Dual-luciferase reporter assays reveals miR-124-3p is involved in the crossregulation between NEAT1 and ATGL. A. Dual-luciferase reporter assays revealed that depletion of NEAT1 in 293 T cells inhibited the luciferase activity of ATGL-WT but not ATGL-MUT. Further, inhibition of miR-124-3p reversed this decrease in luciferase activity for ATGL-WT, but not for ATGL-MUT. Data are expressed as mean ± SD. Statistical significance was concluded at **P < 0.01. NS represents no statistical significance. (TIF 685 kb) [file 12943_2018_838_MOESM12_ESM.tif]

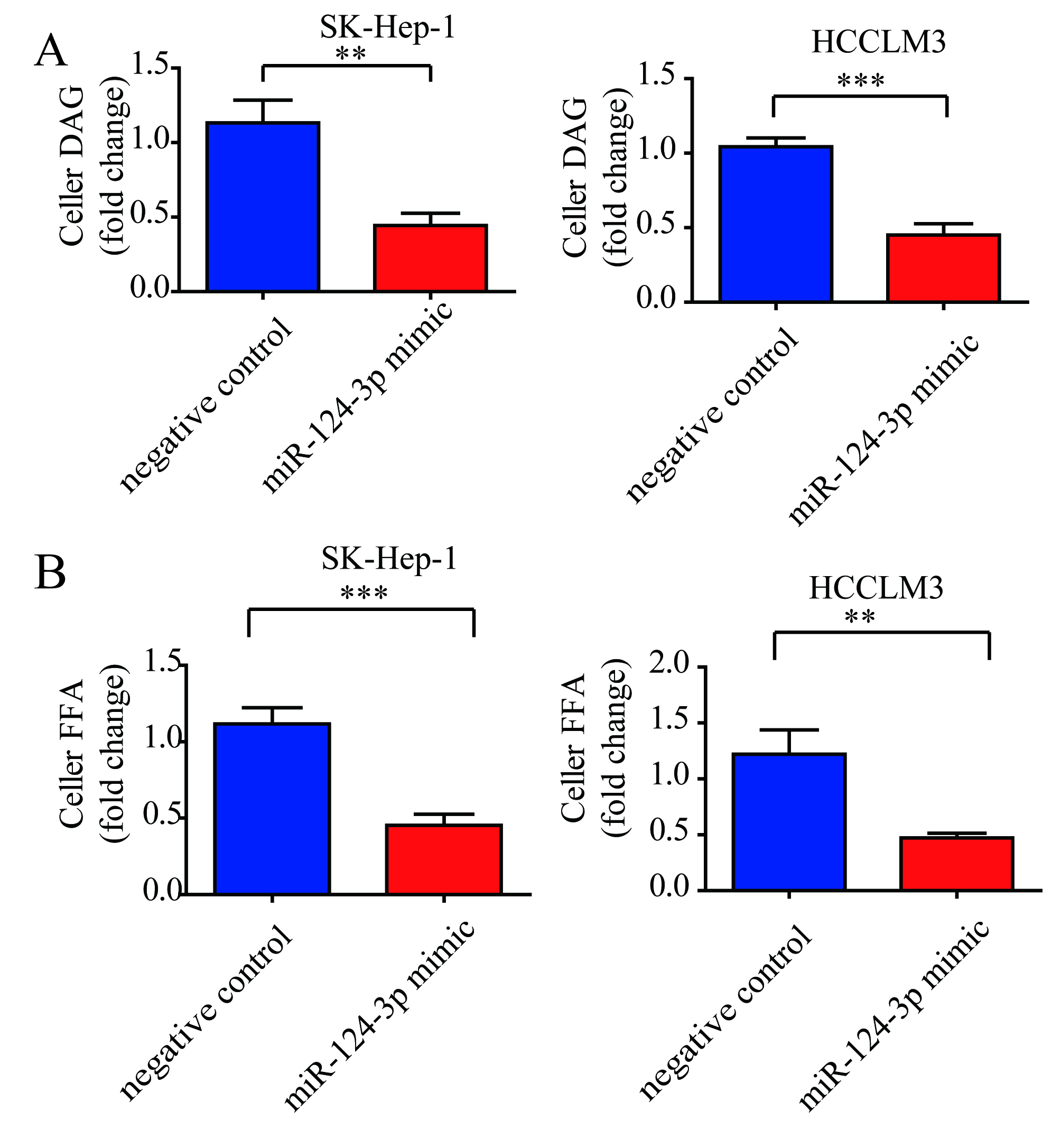

Supplement: Supplementary file 13 — Figure S11. The effect of miR-124-3p on lipolysis. A. Treatment with miR-124-3p mimic decrease intracellular DAG levels in SK-hep-1 and HCCLM3 cells B. Treatment with miR-124-3p mimic decrease intracellular FFA levels in SK-hep-1 and HCCLM3 cells. Data are expressed as mean ± SD of three independent experiments. Statistical significance was concluded at **P < 0.01, ***P < 0.001. (TIF 832 kb) [file 12943_2018_838_MOESM13_ESM.tif]

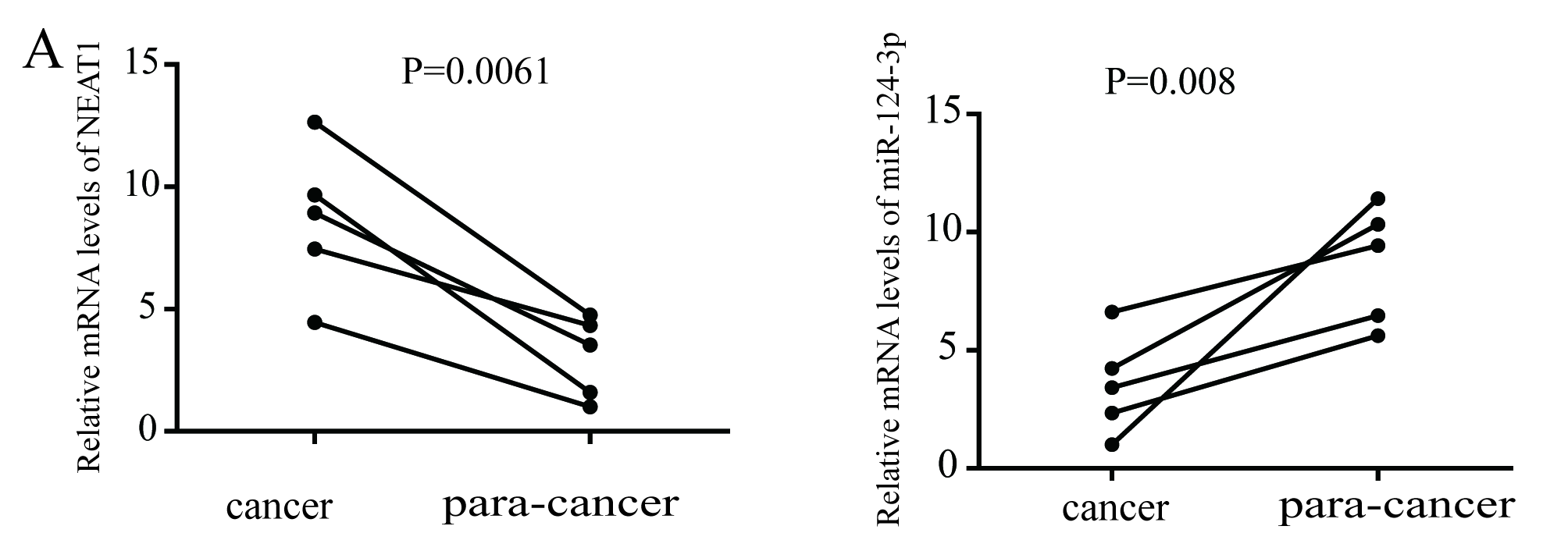

Supplement: Supplementary file 14 — Figure S12. NEAT1 and miR-124-3p mRNA was aberrantly expressed in 5 pairs of HCC and matched non-tumor tissues. A. Real-time PCR analysis of NEAT1 and miR-124-3p expression in five pairs of HCC and matched non-tumor tissues. Data are expressed as mean ± SD of three independent experiments. (TIF 678 kb) [file 12943_2018_838_MOESM14_ESM.tif]
